# Early insight into social network structure predicts climbing the social ladder

**DOI:** 10.1126/sciadv.ads2133

**Published:** 2025-06-20

**Authors:** Isabella C. Aslarus, Jae-Young Son, Alice Xia, Oriel FeldmanHall

**Affiliations:** ^1^ Department of Psychology, Stanford University, Stanford, CA 94305, USA.; ^2^ Department of Cognitive, Linguistic, and Psychological Sciences, Brown University, Providence, RI 02912, USA.; ^3^ Carney Institute for Brain Sciences, Brown University, Providence, RI 02912, USA.

## Abstract

While occupying an influential position within one’s social network brings many advantages, it is unknown how certain individuals rise in social prominence. Leveraging a longitudinal dataset that tracks an entirely new network of college freshmen (*N* = 187), we test whether “climbing the social ladder” depends on knowing how other people are connected to each other. Those who ultimately come to occupy the most influential positions exhibit early and accurate representations of their network’s general, abstract structure (i.e., who belongs to which communities and cliques). In contrast, detailed, granular representations of specific friendships do not translate into gains in social influence over time. Only once the network stabilizes do the most influential individuals exhibit the most accurate representations of specific friendships. These findings reveal that those who climb the social ladder first detect their emerging network’s general structure and then fine-tune their knowledge about individual relationships between their peers as network dynamics settle.

## Teaser

In an evolving social network, accurate knowledge about community structure precedes and predicts becoming influential.

## INTRODUCTION

It has long been theorized that individuals benefit from occupying influential positions in their communities (*1*, *2*). For example, centrally located individuals can draw upon their direct and indirect social connections to spread information and shape norms across their networks (*3*–*6*). The influence that central individuals can exert has been harnessed for powerful interventions that range from reducing bullying (*7*) to supporting financial wellbeing (*3*, *8*) to improving public health (*9*, *10*). Although we now know that an individual’s position within their network confers well-documented social advantages and power (*11*–*19*), it is unknown how individuals succeed at climbing the social ladder to occupy influential positions.

An intuitive and compelling possibility is that some individuals benefit from having better knowledge about the topology of their social networks. Knowing about the vast number of relationships comprising a social network may help individuals to acquire highly central positions (*2*, *12*). The hypothesis that accurate knowledge about one’s network is socially advantageous—which we refer to as “accuracy-as-advantage” (*2*)—is borne out in research illustrating that accurate representations of one’s network (i.e., knowledge about who is connected to whom) are associated with a range of social benefits in real-world networks, such as having more friends (*20*–*24*), gaining better reputations (*25*–*27*), and being sought out for help and advice (*25*). However, the majority of past research measures social networks at a single moment in time, rendering these findings largely descriptive. The result is that little is understood about how exactly individuals come to occupy influential network positions over time. The relationship between accurate network knowledge and social advantage could be explained equally well by an “advantage-as-accuracy” hypothesis, in which advantageous social positions provide better access to information about others’ relationships, enabling people to build more accurate representations of their network. Because cross-sectional network studies cannot distinguish between these possibilities, the mechanisms linking network representation to social advantage remain unclear.

We leverage a unique dataset that remedies these methodological limitations, allowing us to measure the cognitive mechanisms underlying people’s ability to obtain highly central network positions. From the very inception of a network of first-year undergraduates, we longitudinally measure how people are connected to each other, how individuals’ network centrality changes over the course of an academic year, and what knowledge people have about their peers’ social ties. As relationships form and break, the overall structure of a network and the social positions of the people within it are constantly in flux. By tracking these changes over time, we capitalize on the inherently dynamic and evolving nature of social networks to examine whether accurate network representations precede and predict changes in individuals’ network centrality. Specifically, the absence of prior social relationships and entrenched social roles in an entirely new network enables us to decouple representational accuracy from existing social advantages and observe how people climb the social ladder.

In its typical formulation, the accuracy-as-advantage hypothesis supports the idea that an individual can become more influential by having a more accurate representation of the structure of their network. However, existing theory leaves unclear how to operationalize influence because social network science offers many ways to quantify how well-connected an individual is within their network. We therefore provide an empirical test of two especially relevant metrics of network centrality. The most intuitive is degree centrality, which is defined simply as the number of friends an individual has. However, a count of one’s friends is not always the most appropriate measure of social influence. Consider, for example, two individuals who have the same number of friends. If one’s friends are socially isolated, while the other’s friends each have many friends themselves, then the latter is clearly “better connected” in the network at large. This kind of insight is captured by eigenvector centrality (*28*, *29*) and mathematically related measures (*10*), which quantifies how well-connected an individual is to other well-connected peers and serves as a proxy for how much influence one has in the network. Eigenvector centrality, hereafter referred to as influence, is such a socially salient measure that people spontaneously track this information in their peers (*30*, *31*). In addition, highly influential individuals reap distinct benefits from their network positions: They are less likely to be the targets of negative gossip (*16*, *32*), more likely to be perceived positively and hold sway over others’ actions (*8*, *27*, *33*), and better at spreading information through their networks (*3*, *10*). Because influence captures the social advantages of occupying highly central positions beyond simply having a large number of friends, it is especially compelling to understand how people become more influential—not just how people make more friends—over time.

The accuracy-as-advantage hypothesis assumes that “accuracy” refers to representations of specific, pairwise relationships between network members (i.e., “micro-level” knowledge). Past empirical research on accuracy-as-advantage largely reflects this assumption [(*2*, *12*, *26*, *34*–*38*), but see (*39*)]. However, a major (but often overlooked) element of networks is that they also comprise larger meso-level structural elements, such as communities or cliques (i.e., groups of individuals who are more connected to each other than they are to others in the network) (*40*–*44*). These communities form the latent, general structure of the broader network, which may not be explicitly observable. Unlike micro-level knowledge, which reflects the smallest unit of a social network and provides people with greater specificity about the details of individual social ties, meso-level knowledge abstracts across these details to derive an efficient, compressed estimate of what broad regions of the network look like. It is therefore possible that detecting a network’s latent, meso-level structure may prove more efficient and advantageous for climbing the social ladder than knowing about micro-level relationships.

Over the course of an academic year, we measured a social network (*N* = 187) as it emerged (when students arrived on campus without preexisting social ties), dynamically evolved, and then stabilized, allowing us to examine the cognitive mechanisms through which people rise to positions of social influence. We constructed snapshots of the true configuration of a network of first-year undergraduates as they made and broke friendships upon entering college. At multiple time points, we assessed individuals’ knowledge of micro-level relationships and meso-level communities by testing how these two elements of the network’s topology inform their inferences about who is friends with whom in different parts of the network. Last, to test our key hypotheses, we linked people’s micro- and meso-level knowledge to shifts in their network centrality as the network evolved over time.

## RESULTS

### An individual’s influence fluctuates across the network’s evolution

**Fig. 1.**
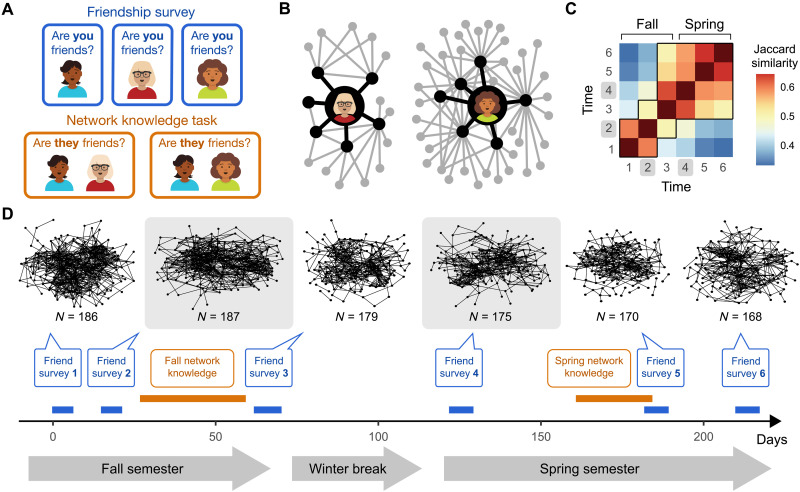
Social network measurements. (**A**) Behavioral tasks. In the friendship surveys (blue), subjects viewed the names and faces of all other subjects in the network and reported whether they were a friend, allowing us to characterize the network’s veridical configuration. In the network knowledge tasks (orange), subjects reported whether they believed that pairs of other network members were friends, enabling the measurement of their knowledge of the network’s structure. (**B**) Influence versus friend count. The neighborhoods of two real subjects in the network illustrate the difference between influence and friend count. These subjects both have six friends (shown in black). However, the individual on the left is much less influential (eigenvector centrality = 0.05) compared to the individual on the right (eigenvector centrality = 0.39). This difference is driven by how well-connected their friends are (friends-of-friends shown in gray). (**C**) Social network stabilization. The emerging network becomes increasingly stable over time, as indicated by higher Jaccard similarity between adjacent time points in the spring versus the fall (e.g., times 4 and 6 versus times 1 and 3). Black outlines highlight all comparisons between two time points with Jaccard similarity > 0.5, revealing that more than half of the friendships present at either time are present at both times. (**D**) Social network configuration over time. Snapshots of the network between October and May show its evolution. Analyses focus on the two time points highlighted in grey boxes: time 2 (measured in October) and time 4 (measured in February), since subjects completed the network knowledge task immediately after completing these friendship surveys, ensuring that subjects’ knowledge was assessed based on the most recent state of the network.

We measured friendships in a social network of first-year undergraduate students throughout the academic year. At each of six data collection timepoints, subjects completed friendship surveys, in which they reported their own friendships with peers in the network (Fig. 1A). These data allowed us to construct snapshots of the true relationships in the network and how they change over time (Fig. 1D). We also used these data to compute two metrics of subjects’ network centrality: degree centrality, or the count of an individual’s friends, and eigenvector centrality, a proxy for social influence that indexes how well-connected an individual is to other well-connected individuals (Fig. 1B). Here, we focus on two particularly important snapshots of the network: during its initial formation (time 2, mid-fall semester; *N* = 187) and again after the network stabilized (time 4, early spring semester; *N* = 176; Fig. 1D).

We first verified that the network itself—and individuals’ positions within it—do indeed change over time. Results reveal a network very much in flux, particularly during its initial formation (Fig. 1C). We assessed how similar the network was across time using Jaccard similarity, which measures the proportion of friendships that are consistent between any two time points, relative to the total number of unique friendships observed across both time points. The three configurations of the network measured in the fall semester were less similar to each other compared to those measured in the spring semester, indicating greater network stability in the spring (Fig. 1C; for more in-depth analysis, see the Supplementary Materials). These observations align with prior research on social network evolution, which documents an early churn of rapidly forming and breaking friendships, followed by greater stability (*45*).

**Fig. 2.**
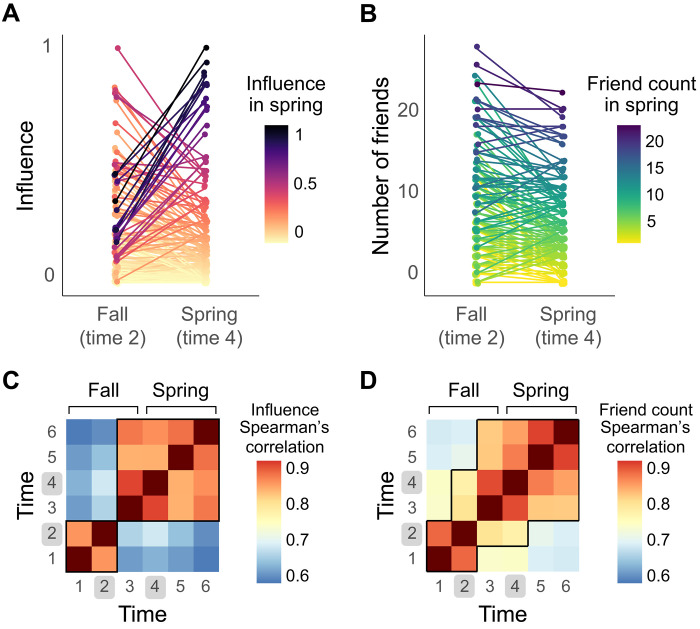
Fluctuations in influence, but not friend count, over time. (**A**) New individuals become influential over time. The most influential subjects in the spring (dark purple) rose up from positions of middling influence in the fall, overtaking those who originally occupied influential positions in the fall (magenta). (**B**) Individuals have stable friend count over time. On average, subjects with the most friends in the spring also had the most friends several months earlier, in the Fall. (**C**) Influence shifts and then stabilizes. Spearman rank correlations of individuals’ influence at every time point reveal that who is influential shifts dramatically in the fall, between times 2 and 3, and then remains very stable throughout the remainder of the academic year. (**D**) Friend count remains relatively stable over time. Spearman’s rank correlations of individuals’ friend counts reveal relatively greater similarity between more distant time points (i.e., between the fall and spring). Black outlines highlight all comparisons between two time points with Spearman’s rho > 0.75.

Next, we tested whether an individual’s centrality undergoes major changes as the network evolves. We focused on the second and fourth time points, which correspond to when we administered network knowledge tasks to probe subjects’ representations of the network in the fall and spring (Fig. 1D). In both the emerging network in the fall and the more stable network in the spring, we ranked individuals by their influence and friend count. Most of the highly influential individuals in the fall did not maintain their positions of influence (Fig. 2A). By the time the network stabilized in the spring, a different cohort of individuals occupied 90% of the top 20 most influential positions (Fig. 2A). Spearman rank correlations of individuals’ influence at every time point confirmed that one’s influence varies considerably between the inception of the network and the next several months; however, starting at the end of the fall semester, influence remains highly correlated across time until the end of the academic year, suggesting greater stability of network centrality as the network matures (Fig. 2C). While we observed a substantial reconfiguration of influence within the network, we did not observe these dramatic changes in friend count. In general, the individuals who had the most friends in the fall continued to have the most friends in the spring (Fig. 2B), and friend count was more stable across time compared to influence (Fig. 2D). Thus, in an evolving network, influence appears to be malleable, with certain individuals moving in and out of highly influential positions. In contrast, the number of friends remains stable over time, suggesting that large shifts in social prominence are specific to influence.

### Early accurate meso-level representation precedes rising influence

While influence (and not friend count) appears to be a dynamic and evolving feature of a social network, it remains unclear what enables people to occupy highly central positions by climbing the social ladder. Might the individuals who start off with middling influence and become highly influential by the end of the academic year do so by leveraging accurate representations of how the network is structured? We tested this by measuring network representations in a subset of all subjects in the fall and spring (*N* = 100 and 80, respectively) using our network knowledge task, in which subjects reported their beliefs about whether pairs of other network members are friends (Fig. 1A). We examined subjects’ inferences about social ties both within and beyond their immediate social circles, thus probing their knowledge about the topology of the network at large, including in parts of the network that were not well known to the subject.

**Fig. 3.**
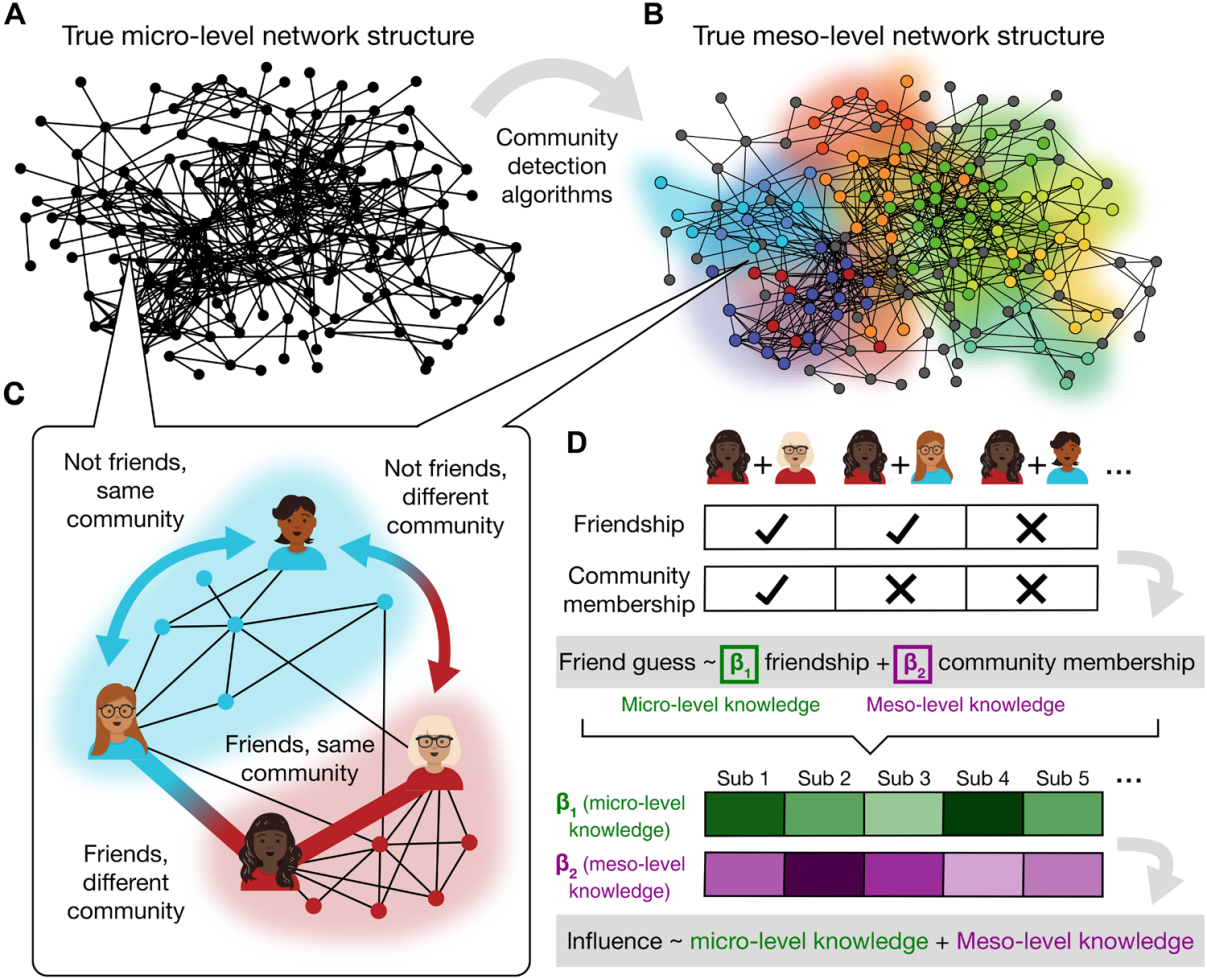
Micro-level versus meso-level network structure. (**A**) Micro-level network structure. The true (veridical) micro-level relationships that comprise the social network. (**B**) Meso-level network structure. Clusters of micro-level relationships form larger communities, which make up the veridical meso-level structure of the social network. (**C**) Toy illustration of micro- and meso-level social ties. In addition to their micro-level relationship (i.e., whether they are friends or not), two network members can have a meso-level social tie, which indicates whether they belong to the same community or not. These constructs are overlapping but distinct, since not all friends are members of the same community and not all members of a community are friends. (**D**) Computing micro-level versus meso-level knowledge. Subject-level logistic regressions predict friendship inferences (measured in the network knowledge task) using two predictors taken from the data in the friendship survey: veridical friendship (micro-level) and veridical community membership (meso-level). Beta coefficients from these regressions estimate the extent to which each subject relies on knowledge of specific friendships versus broader communities. These subject-level beta coefficients are then extracted and used as predictors in group-level analyses, such as predicting subjects’ influence.

Given our hypothesis that individuals may benefit more from meso-level knowledge about the broader communities in a network, rather than micro-level knowledge about specific social ties, we computed the extent to which each subject’s representation of the network reflected micro-level network structure (Fig. 3A) versus meso-level network structure (Fig. 3B). To do so, we first identified the network’s meso-level communities using data-driven cluster detection algorithms (see Materials and Methods). Next, we estimated each subject’s micro- and meso-level knowledge by taking advantage of the fact that not all friends are in the same community and not all members of a community are friends (Fig. 3C). We reasoned that a subject whose network representation prioritizes meso-level network structure would be more likely to both identify true friendships and produce false positives within the same community, while missing true friendships and avoiding false positives that span separate communities. This subject’s inferences about others’ friendships should therefore reflect greater reliance on information about community membership. To evaluate this, we first quantified relative knowledge of micro-level versus meso-level network structure by running subject-specific logistic regressions (Eq. 1). The purpose of these regressions was to estimate how strongly each subject’s beliefs about their peers’ friendships are influenced by true pairwise friendships versus the broader communities to which their peers belong (Fig. 3D, top portion of the panel).

**Fig. 4.**
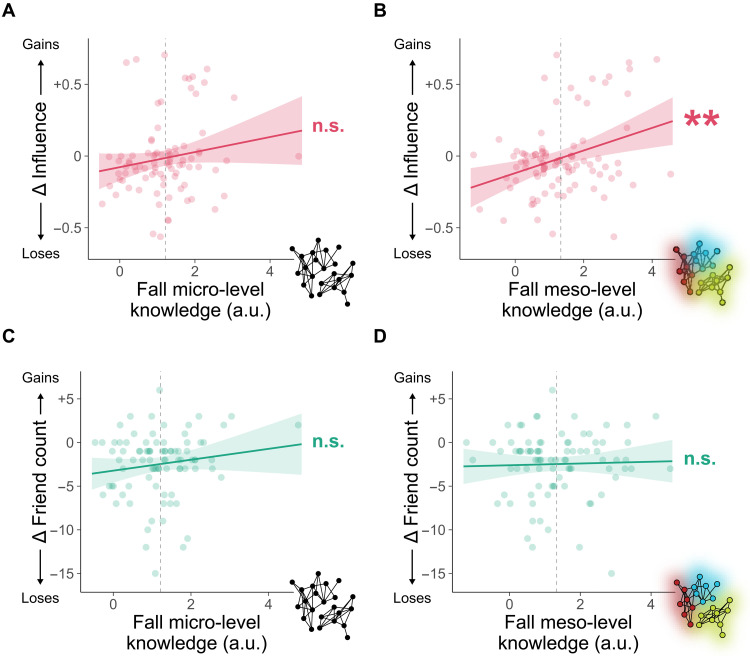
Accurate meso-level representations boost influence but not friend count. (**A**) Rising influence, from the fall to spring, is not related to micro-level knowledge (i.e., specific pairwise relationships in the network). (**B**) Inferences about others’ friendships based on meso-level network structure (i.e., community membership) predict greater influence over time. (**C** and **D**) Changes in friend count between the fall and spring are not predicted by either micro-level knowledge (C) or meso-level knowledge (D). Dashed lines represent the mean beta coefficient (i.e., mean knowledge) across subjects. Error ribbons represent the 95% CI. **P < 0.01. a.u., arbitrary unit.

We extracted subject-specific beta coefficients, which we treat as measures of micro- and meso-level knowledge, as they represent the extent to which each subject’s representation relies on micro-level friendships versus meso-level communities. These coefficients were then used as predictors in group-level regressions (Fig. 3D, bottom portion of the panel). This allowed us to test, at the group level, whether these two types of knowledge lead to increasing influence or friend count over time (Eq. 2; Table 1). Results reveal that the more an individual exhibits knowledge of latent, meso-level network structure in the fall, the more likely they are to become influential by the spring [β = 0.08, 95% confidence interval (CI) = [0.03, 0.13], *P* = 0.001; Table 1, M1], an effect that robustly persists when controlling for how outgoing a person is (i.e., extroversion) and how many friends they had in the fall (Fig. 4B; β = 0.08, 95% CI = [0.03, 0.13], *P* = 0.002; Table 1, M2). In contrast, specific, micro-level knowledge did not predict increasing influence over time when accounting for extroversion or friend count (Fig. 4A; β = 0.05, 95% CI = [−0.01, 0.12], *P* = 0.106; Table 1, M2) and had only a modest effect without controlling for extroversion or friend count (β = 0.06, 95% CI = [0.002, 0.13], *P* = 0.042; Table 1, M1). Put simply, rising influence appears to follow from early knowledge of the communities that form the underlying latent structure of one’s network—not individual differences in personality, early popularity, or one’s micro-level knowledge of specific relationships.

**Table 1.** Results of models predicting changes in influence and friend count (outcome variables) between fall and spring. Specifications for each model shown above model output. Beta coefficients (β), 95% CIs, and *P* values shown for each predictor. *P < 0.05, **P < 0.01, and ***P < 0.001

Outcome variable	Predictor	β	95% CI (lower, upper)	*P* value
M1: *change in influence* ~ β_0_ + β_1_ *fall micro-level knowledge* + β_2_ *fall meso-level knowledge*				
Δ Influence	Intercept	−0.20	(−0.31, −0.09)	7.6 × 10^−4^***
	Fall micro	0.06	(0.002, 0.13)	0.042*
	Fall meso	0.08	(0.03, 0.13)	0.001**
M2: *change in influence* ~ β_0_ + β_1_ *fall micro-level knowledge* + β_2_ *fall meso-level knowledge* + β_3_ *fall friend count* + β_4_ *fall extroversion*				
Δ Influence	Intercept	−0.22	(−0.46, 0.02)	0.069
	Fall micro	0.05	(−0.01, 0.12)	0.106
	Fall meso	0.08	(0.03, 0.13)	0.002**
	Fall friend count	−0.01	(−0.02, 0.003)	0.153
	Extroversion	0.004	(−0.004, 0.01)	0.322
M3: *change in friend count* ~ β_0_ + β_1_ *fall micro-level knowledge* + β_2_ *fall meso-level knowledge*				
Δ Friend count	Intercept	−3.36	(−5.01, −1.70)	1.2 × 10^−4^***
	Fall micro	0.63	(−0.28, 1.54)	0.171
	Fall meso	0.08	(−0.62, 0.77)	0.829

Gaining friends over time was not associated with accurate network representation, as greater friend count in the spring was not predicted by either micro-level knowledge (Fig. 4C; β = 0.63, 95% CI = [−0.28, 1.54], *P* = 0.171; Table 1, M3) or meso-level knowledge (Fig. 4D; β = 0.08, 95% CI = [−0.62, 0.77], *P* = 0.829; Table 1, M3) in the fall. Thus, the ability to detect meso-level communities plays a unique role in helping people gain influence—but not make more friends—over time. We verify the robustness of this finding with an additional test of our hypothesis, in which we simulate control and treatment groups that are matched on influence but differ in their early network knowledge, which fully replicates the results of our main analysis (Supplementary Materials). These results provide evidence in favor of the accuracy-as-advantage hypothesis, offering additional theoretical insight into which kinds of knowledge enable an individual to accrue social advantages.

### Micro-level knowledge is associated with current influence once the network stabilizes

Past research on accuracy-as-advantage has focused on the social benefits of accurate representations of pairwise friendships, which seems to conflict with our finding that it is knowledge about meso-level network structure, and not micro-level relationships, that predicts rising influence. However, previous research has focused on the current social advantages conferred by accurate knowledge in a stable network, at a single snapshot in time (*20*–*26*). It may be possible to reconcile this apparent inconsistency by testing whether micro-level knowledge is linked to current influence at a given time point in our dataset, both before and after the network stabilizes. We used the same subject-specific estimates of micro- and meso-level knowledge in the fall and spring to predict current influence and friend count (Eq. 3; Table 2).

**Table 2.** Results of models predicting current influence and friend count (outcome variables) in fall and spring. Specifications for each model shown above model output. Beta coefficients (β), 95% CIs, and *P* values shown for each predictor. **P < 0.01, and ***P < 0.001.

Outcome variable	Predictor	β	95% CI (lower, upper)	*P* value
M1: *fall influence* ~ β_0_ + β_1_ *fall micro-level knowledge* + β_2_ *fall meso-level knowledge*				
Fall influence	Intercept	0.31	(0.22, 0.40)	1.2 × 10^−9^***
	Fall micro	−0.04	(−0.09, 0.003)	0.069
	Fall meso	−0.01	(−0.05, 0.03)	0.653
M2: *spring influence* ~ β_0_ + β_1_ *spring micro-level knowledge* + β_2_ *spring meso-level knowledge*				
Spring influence	Intercept	−0.01	(−0.15, 0.13)	0.870
	Spring micro	0.14	(0.04, 0.24)	0.006**
	Spring meso	0.06	(−0.02, 0.13)	0.131
M3: *fall friend count* ~ β_0_ + β_1_ *fall micro-level knowledge* + β_2_ *fall meso-level knowledge*				
Fall friend count	Intercept	11.06	(8.43, 13.69)	7.5 × 10^−13^***
	Fall micro	−1.13	(−2.51, 0.25)	0.107
	Fall meso	0.39	(−0.69, 1.47)	0.471
M4: *spring friend count* ~ β_0_ + β_1_ *spring micro-level knowledge* + β_2_ *spring meso-level knowledge*				
Spring friend count	Intercept	5.11	(2.17, 8.05)	9.0 × 10^−4^***
	Spring micro	1.31	(−0.71, 3.32)	0.201
	Spring meso	0.80	(−0.73, 2.32)	0.302

**Fig. 5.**
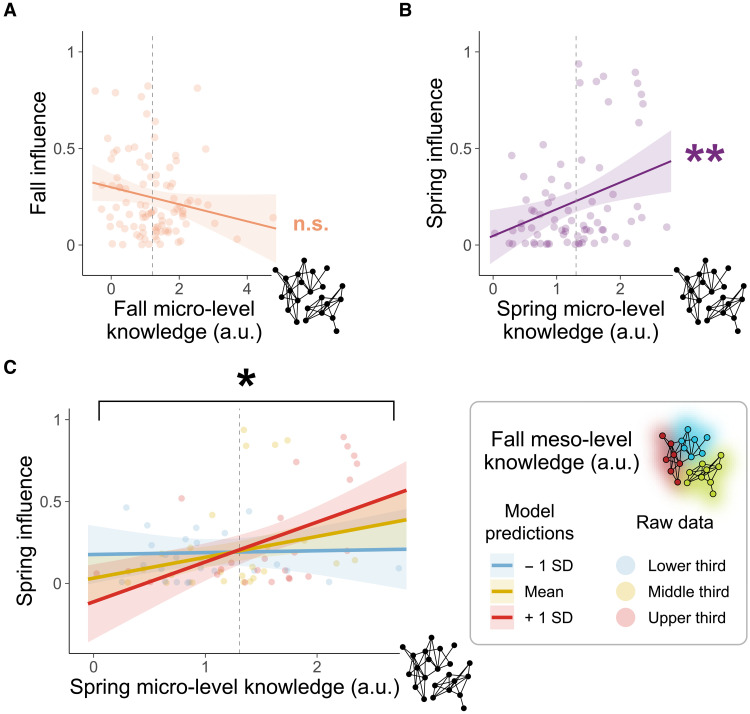
Stable influence is linked to early meso-level and late micro-level knowledge. (**A**) In the fall, micro-level knowledge does not predict current influence. (**B**) In the spring, influence is uniquely associated with knowledge about the true micro-level relationships in the network. (**C**) Fall meso-level knowledge interacts with spring micro-level knowledge to predict spring influence. Greater influence is associated with accurate representation of communities in the fall and accurate representation of pairwise friendships in the spring. Dashed lines represent the mean beta coefficient (i.e., mean knowledge) across subjects. Error ribbons represent the 95% CI. *P < 0.05 and **P < 0.01. a.u., arbitrary unit.

When the network was highly unstable in the fall—and many of the most influential individuals went on to lose their influence by spring (Fig. 2A)—current influence was not associated with current micro-level (Fig. 5A; β = −0.04, 95% CI = [−0.09, 0.003], *P* = 0.069; Table 2, M1) or meso-level (β = −0.01, 95% CI = [−0.05, 0.03], *P* = 0.653; Table 2, M1) knowledge. We observed a similar pattern of results for friend count (micro-level β = −1.13, 95% CI = [−2.51, 0.25], *P* = 0.107; meso-level β = 0.39, 95% CI = [−0.69, 1.47], *P* = 0.471; Table 2, M3). Essentially, we found no relationship between early network position and early representation of the social network; if anything, greater influence trended toward worse knowledge (Fig. 5A and Table 2, M1). This early decoupling of accurate knowledge and social advantage, together with the finding that early meso-level knowledge predicts rising influence (Fig. 4B), lends further support to the accuracy-as-advantage hypothesis (as opposed to advantage-as-accuracy). By showing that accurate knowledge precedes climbing the social ladder, our results support the hypothesis that knowledge about the broader structure of one’s social network confers social advantages. Furthermore, several months later in the spring, accurate micro-level knowledge comes to strongly predict current influence (Fig. 5B; β = 0.14, 95% CI = [0.04, 0.24], *P* = 0.006; Table 2, M2), whereas we no longer observe a strong relationship between influence and knowledge of meso-level network structure (β = 0.06, 95% CI = [−0.02, 0.13], *P* = 0.131; Table 2, M2). Thus, once a social network has stabilized, occupying a position of ongoing influence is associated with exhibiting a fine-grained representation of pairwise friendships, consistent with previous cross-sectional research (*2*). As before, friend count in spring was not associated with current knowledge of either micro-level (β = 1.31, 95% CI = [−0.71, 3.32], *P* = 0.201; Table 2, M4) or meso-level (β = 0.80, 95% CI = [−0.73, 2.32], *P* = 0.302; Table 2, M4) social ties.

The fact that early meso-level knowledge predicts rising influence (Fig. 4B), while micro-level knowledge is only associated with current influence once the network stabilizes (Fig. 5B), implies that different types of representation have distinct contributions to gaining influence. To directly test whether eventual influence relies on both early and late representations, we predicted late influence in the spring by interacting early meso- and late micro-level knowledge (Eq. 4; Table 3). Results show that the most influential individuals at the end of the year were those who had both accurate knowledge about latent communities during the network’s formation and accurate knowledge about specific relationships once the network stabilized (Fig. 5C; β = 0.10, 95% CI = [0.02, 0.18], *P* = 0.014; Table 3, M1). This effect held when controlling for all other combinations of early and late knowledge (β = 0.10, 95% CI = [0.01, 0.20], *P* = 0.028; Table 3, M2), none of which predicted spring influence (Table 3, M2). Thus, one’s eventual (and stable) influence is jointly determined by the ability to initially detect the early network’s meso-level structure and, later, fine-tune one’s representation of micro-level relationships as the network stabilizes.

**Table 3.** Results of models predicting spring influence (outcome variable) using both fall and spring knowledge (predictors). Specifications for each model shown above model output. Beta coefficients (β), 95% CIs, and *P* values shown for each predictor. *P < 0.05.

Outcome variable	Predictor	β	95% CI (lower, upper)	*P* value
M1: *spring influence* ~ β_0_ + β_1_ *fall meso-level knowledge* + β_2_ *spring micro-level knowledge* + β_3_ (*fall meso-level knowledge* × *spring micro-level knowledge*)				
Spring influence	Intercept	0.20	(0.01, 0.39)	0.041*
	Fall meso	−0.13	(−0.26, 0.003)	0.056
	Spring micro	−0.01	(−0.16, 0.14)	0.909
	Fall meso × spring micro	0.10	(0.02, 0.18)	0.014*
M2: *spring influence* ~ β_0_ + β_1_ *fall micro-level knowledge* + β_2_ *fall meso-level knowledge* + β_3_ *spring micro-level knowledge* + β_4_ *spring meso-level knowledge* + β_5_ (*fall meso-level knowledge* × *spring micro-level knowledge*) + β_6_ (*fall micro-level knowledge* × *spring micro-level knowledge*) + β_7_ (*fall micro-level knowledge* × *spring meso-level knowledge*) + β_8_ (*fall meso-level knowledge* × *spring meso-level knowledge*)				
Spring influence	Intercept	0.29	(0.02, 0.56)	0.037*
	Fall micro	−0.04	(−0.21, 0.13)	0.644
	Fall meso	−0.15	(−0.30, −0.02)	0.030*
	Spring micro	−0.18	(−0.42, 0.06)	0.144
	Spring meso	0.03	(−0.17, 0.22)	0.781
	Fall meso × spring micro	0.10	(0.01, 0.20)	0.028*
	Fall micro × spring micro	0.11	(−0.02, 0.25)	0.096
	Fall micro × spring meso	−0.03	(−0.14, 0.07)	0.507
	Fall meso × spring meso	0.02	(−0.05, 0.10)	0.529

## DISCUSSION

What enables people to rise to prominent positions within their social network? Rewriting the conventional wisdom that “it’s not what you know, but who you know” that determines social success, our results suggest that “what you know” about your network shapes “who you know.” We show that in an emerging network, the people who exhibit early and accurate representations of the network’s topology are the ones who ultimately climb to the top of the social ladder. Ascending to a more influential position is solely predicted by early knowledge about the social network’s meso-level structure (i.e., communities and cliques). However, as the network evolves, we observe changes in which type of network representation facilitates greater influence. Once the network stabilizes, influence becomes tightly yoked to holding accurate micro-level representations of specific relationships, especially for individuals who also had accurate representations of meso-level communities when the network was first forming. Thus, detecting the latent community structure of an emerging network is associated with accruing influence over time, and those who ultimately occupy positions of influence go on to develop particularly accurate knowledge about the specific relationships that comprise their networks.

Our results indicate that knowing about meso-level network structure is an important contributor to rising influence over time, which challenges dominant theoretical assumptions underlying the accuracy-as-advantage hypothesis. To date, the assumption has been that adaptive social behavior is aided by accurate representations of specific, pairwise friendships in the network (*2*, *12*, *26*, *34*–*38*). According to this account, evidence of community detection—for example, inferring that members of the same community are friends even if they do not report a friendship or missing friendships between members of different communities—is interpreted as mistakes or cognitive shortcuts that only diminish the accuracy of one’s network representation (*12*, *41*, *46*–*48*). Our findings suggest otherwise. The ability to accurately detect a network’s latent structure seems to be a major source of social advantage, although such a representation might be considered coarse and riddled with (systematic) errors. Thus, these results suggest a reframing of the accuracy-as-advantage hypothesis, challenging how the field often defines and measures the accuracy of social network representations. For instance, mixed findings in the literature related to accuracy-as-advantage (*37*, *49*–*51*) may be attributable, in part, to the fact that only micro-level knowledge is considered. In these cases, accurate meso-level knowledge would not be detectable and could even manifest as diminished accuracy at the micro-level.

Why might individuals’ trajectories toward (or away from) influential network positions be uniquely linked to their early representations of meso-level network structure, but not micro-level relationships? When considering what behavioral affordances are conferred by various kinds of network representation, there are a few theoretical disadvantages associated with only holding micro-level knowledge of relationships. Maintaining and using an accurate micro-level representation requires encoding, retrieving, and computing over many pairs of network members. This poses a formidable cognitive challenge because the number of possible pairwise relationships combinatorically explodes with the number of individuals in the network. Furthermore, a micro-level representation typically necessitates computationally expensive processes such as tree search to make useful inferences about the network (e.g., how information flows), as there are a vast number of potential pathways that could connect any given pair of individuals. In contrast, at the meso-level, accurate knowledge and inferences can be achieved through substantially reduced demands on information storage and computation by leveraging a compressed representation of the broader communities in the network (*46*, *52*–*55*). Such an abstract meso-level representation therefore facilitates a range of cognitive processes that can support navigation in a vast and dynamic social landscape: Inferring how information might flow through a network, predicting the existence of unobserved but structurally probable relationships, and even anticipating the formation of new relationships that do not yet exist (*30*, *56*–*58*). Our results support the idea that social advantages, such as those associated with influence, are linked to the ability to leverage compressed representations that can be flexibly used in multiple different contexts—a hallmark of higher-order cognition where learning latent structure affords flexible generalization beyond the immediate here and now (*57*–*63*).

Across all our analyses, we found substantial fluctuations in influence (i.e., eigenvector centrality), but not simple friend count (i.e., degree centrality). This suggests that influence is a more malleable—and likely critical—feature of a social network’s structure. Our findings further suggest that it is important to consider how one is embedded in the network at large, as friend count measures one’s centrality in a relatively contextless vacuum and does not take stock of the larger social ecosystem. In the same vein as other research on the personal and professional advantages that come from one’s weak ties (versus strong ties, e.g., acquaintances versus friends) (*64*), this work highlights the importance of indirect ties (versus direct ties), as captured by eigenvector centrality.

Social life is ever-changing, which makes longitudinal network analysis critical for understanding cognitive processes as they unfold in the wild. By adopting a longitudinal perspective, we can resolve apparent conflicts in cross-sectional findings. For example, although accurate network knowledge has previously been linked to current network centrality (*2*, *20*, *21*, *23*, *24*), we show that this relationship only holds in a stable network, but not one that is fluctuating. Our approach also supports directional and mechanistic claims, such as our finding that early knowledge precedes the social advantages that it confers and predicts rising to a more influential position over time. Furthermore, the dynamic nature of social networks undermines normative claims about adaptive social behavior that are based on a single snapshot in time because optimal behavior can differ dramatically in evolving, complex systems. For example, the relative benefits of micro-level versus meso-level knowledge may depend on the age and stability of a social network. In a brand new social network that is unstable and uncharted, people can rapidly build meso-level knowledge by inferring latent structure from sparse observations of others’ social interactions (*58*). While a micro-level representation would afford greater specificity, it likely requires piecing together many more direct observations over time, making it slower to build and less adaptable in a changing environment. Thus, leveraging meso-level network structure may be a better strategy in an emerging network, while micro-level knowledge becomes increasingly valuable over time as the network stabilizes. Our results mirror this theoretical shift in the utility of micro-level versus meso-level knowledge over time. This insight is made possible by taking a longitudinal approach to characterize a real-world social network, which further highlights unexplored territory for future research about how adaptive social behavior might differ across time and context, and how people might flexibly tune their behavior to the dynamics of their ever-changing social environment.

Our study leaves open several questions about the generalizability of its findings, which provides ample opportunity for future work. Because our subjects were all first-year undergraduates, it remains unknown whether the temporal dynamics of social network knowledge and centrality operate the same way in different contexts (e.g., other age ranges and other academic, residential, professional, and/or friendship networks). Furthermore, we used the testbed of a brand-new network that evolves and stabilizes to suggest that the utility of micro-level versus meso-level knowledge may map onto the stability of a social network. While the formation of a completely new network is relatively rare, existing networks can also experience shifts in stability (e.g., when two companies merge, or when a key member of a friend group moves away). Future work should therefore examine whether the patterns observed in this emerging social network can generalize to an existing social network undergoing changes in stability.

Last, it remains unknown precisely how network knowledge contributes to rising influence. We propose that knowledge of individual friendships and the broader community structure within a network helps individuals accrue influence, but we note that alternative explanations are possible. For instance, it could be the case that an unobserved third variable might simultaneously enhance people’s access to information about network structure and increase opportunities to befriend their peers (e.g., living in a more central location in the dorm’s spatial layout, or being enrolled in the same classes as other network members). It is also possible that an additional variable could explain heterogeneity in eventual influence among individuals who have similar knowledge at the network’s inception. While we control for some personality-level confounds such as extroversion, in a naturalistic field study, it is difficult to account for every possible confound or alternative explanation. Future work should examine whether network knowledge predicts specific social behaviors that, in turn, predict influence, and should evaluate this alongside alternative explanations for the link between network knowledge and influence.

Social networks are dense, interconnected, and evolving, which makes it uniquely challenging to build and maintain the social knowledge that is vital for successfully navigating the social world. Here, we test whether mental representations of one’s social network are a directional mechanism that predicts one’s social standing over time. We show that early knowledge about latent network structure predicts climbing the social ladder. This research lays the groundwork for understanding real-world social cognition and behavior—and its consequences for social success and well-being—outside the laboratory, embedded within all the complexities of daily life.

## MATERIALS AND METHODS

### Subjects

Because we were interested in measuring the development of a social network from its inception, only first-year undergraduate students at Brown University were eligible for our study. We recruited 198 subjects from three residential communities, but two subjects were later deemed ineligible because they did not meet these eligibility criteria, resulting in a final sample of 196 subjects (103 female, 4 other gender; 146 non-white or mixed race; mean age = 18.06, SD ± 0.42). Within this sample, 187 subjects completed the second friendship survey (of the six) in mid-fall (100 female, 4 other gender; 140 non-white or mixed race; mean age = 18.06, SD ± 0.42), and 176 subjects completed the fourth friendship survey in early spring, but one subject was excluded because they did not follow the survey instructions (96 female, 3 other gender; 133 non-white or mixed race; mean age = 18.06, SD ± 0.42). We use these two friendship surveys as our measure of the network’s ground-truth configuration in key analyses comparing subjects’ network knowledge in the fall and spring.

The network knowledge task was administered immediately after each of the two friendship surveys. In the fall, the network knowledge task was completed by 100 subjects (54 female, 2 other gender; 79 non-white or mixed race; mean age = 18.06, SD ± 0.42). In the spring, it was completed by 80 of those 100 subjects (45 female; 62 non-white or mixed race; mean age = 18.05, SD ± 0.45). All subjects were paid $10 per hour for online surveys and $15 per hour for in-laboratory study sessions. To retain participation throughout the academic year, subjects who completed all online sessions earned a bonus of $50, and subjects who completed all in-laboratory sessions earned an additional bonus of $50. All procedures were approved by Brown University’s Institutional Review Board (protocol 1911002585), and informed consent was obtained from all subjects. For 17-year-old minors, informed consent was obtained from their legal guardians, and assent was obtained from the subjects.

### Friendship surveys

Six times throughout the academic year (three per semester), subjects completed an online questionnaire (friendship survey) in which they reported their friendship status with all other subjects (Fig. 1A). Subjects received instructions adapted from Parkinson *et al.* (*31*), which read: “Your friends are defined as the people with whom you like to spend your free time. Since you arrived at Brown, who are the people you have been with most often for informal social activities, such as going out to lunch, dinner, drinks, films, visiting one another’s rooms, and so on?” In the first two friendship surveys, subjects indicated whether each person was a friend or not. Starting from the third friendship survey, subjects were required to provide additional ratings for non-friends that consisted of “Acquaintance,” “Recognize,” and “Do not know.” Following conventional practices in social network analysis (*65*), we determined that a friendship existed if two subjects mutually reported each other as “Friends.” These self-reported friendships, or micro-level social ties, were used to construct the network’s true micro-level configuration at different points in time (Fig. 1D).

### Network centrality

Using these snapshots of the network, we characterized each subject’s network centrality at every timepoint (Fig. 1B) using the R packages tidygraph and igraph (*66*, *67*). Degree centrality (which we refer to as “friend count”) is defined as an individual’s total number of mutual friendships. For individuals who were included in the network of mutual friendships (i.e., anyone who had at least one mutual friend), fall friend count ranged from 1 to 28 (mean = 8.63, SD ± 5.98), and spring friend count ranged from 1 to 23 (mean = 6.77, SD ± 4.96). To assess influence, we computed each subject’s eigenvector centrality, a measure bounded between 0 (low influence) and 1 (high influence) that captures how well-connected one is to well-connected peers, relative to everyone else in the network. In the fall, influence ranged from 0.0003 to 1 (mean = 0.20, SD ± 0.20), and in the spring, influence also ranged from 0.0003 to 1 (mean = 0.18, SD ± 0.23). We quantified changes in these two centrality measures over time by simply subtracting a subject’s earlier measure from a later one, such that a positive difference score indicates increasing centrality over time. Change in friend count ranged from −15 to 6 (mean = −2.20, SD ± 3.59) and change in influence ranged from −0.56 to 0.70 (mean = −0.03, SD ± 0.23).

### Network stability

To quantify network stability (Fig. 1C), we used the Jaccard similarity index, which is defined as the ratio of the union of two sets to the intersection of those sets. In the case of a social network, each set consists of all mutual friendships (i.e., edges) in the network at a given time point. To analyze how stable the distribution of centrality in the network was over time (Fig. 2, C and D), we computed the Spearman’s rank correlation of subjects’ centrality (influence and friend count) between every pair of time points.

### Micro-level and meso-level network structure

From each snapshot of the network, generated from the friendship surveys, we quantified both the self-reported relationships between subjects (micro-level structure; Fig. 3A) and the communities to which they belong (meso-level structure; Fig. 3B). Veridical micro-level network structure was defined by all the mutual friendships reported in a friendship survey. In other words, a micro-level social tie existed between a pair of subjects if (and only if) both claimed to be friends with each other.

To estimate the network’s veridical meso-level structure, we used data-driven cluster detection algorithms from the R package igraph (*67*) to identify latent communities in the network (i.e., densely-interconnected groups of network members) at each time point (Fig. 3B). There is little theoretical agreement about how to define a social community, so to remain agnostic to any particular community detection algorithm, we ultimately assigned subjects to communities using the consensus (i.e., the intersection) of four common algorithms in network analysis: the map equation, which is based on probabilistic information flow (*53*, *68*); an algorithm that uses edges with high betweenness to identify community boundaries (*69*); a procedure for directly optimizing a network’s modularity (*70*); and an algorithm that defines communities as regions where short random walks tend to get trapped (*71*). A group of subjects was considered to comprise a community if (and only if) all four community detection algorithms assigned at least five subjects to the same community. By taking the intersection of the communities detected by four algorithms, our “consensus” communities represent the most robust clusters in the network, which are agnostic about the algorithm used for cluster detection because they have been independently identified by all four algorithms.

In our consensus measure, 26% of subjects in the fall (49 of the 187) and 35% of subjects in the spring (60 of the 170) were not assigned to any community. The fact that different algorithms could not agree on a community assignment for a subset of network members aligns with the reality of social life: Some individuals do not belong to a distinct cluster of friends but instead straddle multiple communities or sit on the fringes of the network.

On both a theoretical and empirical level, we note that micro- and meso-level social ties within the network are distinct constructs (Fig. 3C), as not all friends are in the same community (in the fall, only 49% of pairs connected by a micro-level social tie also had a meso-level tie, and 51% in the spring), and not all members of a community are friends (in both the fall and spring, only 43% of those with a meso-level social tie also had a micro-level tie).

### Network knowledge task

#### Timeline

At three time points throughout the academic year, a further subset of subjects completed the network knowledge task, in which they reported their beliefs about whether pairs of other network members were friends (Fig. 1A). Here, we focus on two relevant time points: the first network knowledge task, which was administered during the network’s initial formation (mid-fall semester), and the second network knowledge task, which was administered after the network had stabilized (early spring semester). To test the robustness of our results, we collected a third wave of task data in late spring, which replicates results from earlier in the spring semester (Supplementary Materials). Each wave of network knowledge tasks was completed within ~1 month of a friendship survey (in which subjects were queried about their own friendships using a roster-based method), and we used the immediately preceding friendship survey to define the ground-truth network that was then used to determine subjects’ knowledge about their peers’ friendships in the network knowledge task.

#### Stimulus selection

In the network knowledge task, each subject was asked to report (or infer) the friendship status of every possible pair of individuals within a sample of 30 other network members (i.e., 435 possible relationships). Because subjects were located in different parts of the network, these samples were specifically tailored to each subject’s network position to include a mix of the subject’s immediate friends and more distant network members. Each subject’s sample consisted of approximately five of the subject’s friends, 10 friends-of-friends, and 15 friends-of-friends-of-friends, although the exact distribution varied and was sometimes constrained by differences in self-reported friendships (e.g., a subject might only report having three immediate friends). We oversampled network members at farther distances from the subject to increase our ability to probe subjects’ representations of more distant regions of the network, about which they are presumably more uncertain. To ensure subjects evaluated both friendships and non-friendships while also avoiding sparsely connected samples with a base rate of true friendship near zero, we ensured whenever possible that each individual in a given sample had at least one friend among the remaining 29 individuals in the sample. To approximately match the base rate of true friendships across subjects’ custom-selected samples, we calculated the average number of true friendships in randomly generated samples across all subjects and then iterated through randomly generated samples for each subject to identify a sample with a total number of true friendships that was closest to the average.

#### Task structure

The network knowledge task consisted of 30 blocks. At the start of each block, subjects were presented with one individual from their custom-selected sample and then reported whether each of the 29 other network members were friends with that individual. Because every individual in the stimulus set was the main target of one block, subjects ultimately provided two responses for every possible pair in the sample, first reporting, for example, whether A is friends with B, then whether B is friends with A. In the first network knowledge task (completed online), subjects reported whether they perceived two people to be “friends” or “not friends.” In the second and third network knowledge tasks (completed in-laboratory), subjects rated the likelihood of friendship between two people on a four-point scale ranging from “very likely” to “very unlikely.” To facilitate logistic regression analysis and comparisons across the three waves of data collection, we binned responses as friends (likely and very likely) and not friends (unlikely and very unlikely).

### Micro- and meso-level knowledge

We individually assessed each subject’s micro- and meso-level knowledge in both the fall and spring (Fig. 3D). To do so, we ran a separate logistic regression for every subject (Eq. 1) to estimate the extent to which each subject’s responses in the network knowledge task (0 = responding “no friendship,” 1 = responding “friendship”; Fig. 1D) reflect inferences based on micro-level versus meso-level network structure. The predictors of this model were: (i) true friendships, i.e., micro-level network structure (self-reported friendships from the friendship survey; 0 = no friendship, 1 = friendship; Fig. 3A) and (ii) true community membership, i.e., meso-level network structure (calculated from friendship survey data by using cluster detection algorithms to determine whether two people belong to the same community, regardless of their friendship status; 0 = different communities, 1 = same community; Fig. 3B)

$$\begin{array}{c} \text{friend guess}∼\beta_{0}+\beta_{1}\;\text{true friendship}\\ \;+\beta_{2}\;\text{true community membership} \end{array}$$ (1)

From these subject-level regressions, we extracted each subject’s beta coefficients. As pairwise friendships reflect micro-level network structure, and communities reflect meso-level structure, these beta coefficients function as subject-level summary statistics that capture the degree to which a subject uses micro-level knowledge (β_1_) and meso-level knowledge (β_2_) when making friend guesses in the network knowledge task. We then use these subject-level beta coefficients as predictors in group-level regressions, allowing us to test, for example, whether these two types of knowledge predict an individual’s change in influence over time. In contrast to other simpler measures of accuracy (e.g., percentage of correctly identified friendships), this method enables us to simultaneously assess micro- and meso-level knowledge by estimating the unique explanatory power of each predictor in the same regression model. We excluded outliers (i.e., beta coefficients more than 3 SDs above or below the mean of the group) and beta coefficients with large standard errors (i.e., more than 3 SDs above or below the mean standard error), indicating model convergence issues for those subjects. As a result, three of the 100 fall subjects and three of the 80 spring subjects were excluded from analysis.

### Effect of early knowledge on network centrality over time

We used linear regression models to test how fall representations predict changes in network centrality between the fall and spring (Table 1). We ran two separate models at the group level to predict changes in two types of network centrality: influence and friend count (Fig. 4, C and D). In both models, the predictors were the estimates of each subject’s micro- and meso-level knowledge in the fall (i.e., the beta coefficients from the subject-level logistic regressions, described above; Eq. 2)

$$\begin{array}{c} \begin{array}{c} \Delta \;{\text{centrality}}_{\left(\text{spring minus fall}\right)}∼\beta_{0}+\beta_{1}\;{\text{micro-level knowledge}}_{\left(\text{fall}\right)}\\ \;+\beta_{2}\;{\text{meso-level knowledge}}_{\left(\text{fall}\right)} \end{array} \end{array}$$ (2)

To account for baseline social connectedness and personality traits that may affect changes in influence, we ran a third model that additionally controlled for subjects’ friend count in the fall and their extroversion (Fig. 4, A and B), which was measured using the Big Five Inventory (*72*, *73*). To separately visualize the effects of micro- and meso-level knowledge, we used the R package ggeffects (*74*) to generate model predictions for our primary predictors of interest, while marginalizing over control predictors (e.g., personality measures).

### Association between current knowledge and network centrality

We took a similar approach to analyze the relationship between current network representations and network centrality in the fall and spring (Table 2). We ran two separate linear regression models at the group level to predict fall influence (Fig. 5A) and fall friend count, each of which had two predictors: subjects’ micro-level knowledge and meso-level knowledge in the Fall (i.e., the beta coefficients from the subject-level logistic regressions, described above in Eq. 1). We repeated this process to predict spring influence (Fig. 5B) and spring friend count using spring knowledge. All four regression equations followed the same structure (Eq. 3)

$$\begin{array}{c} \begin{array}{c} {\text{centrality}}_{\left(\text{fall or spring}\right)}∼\beta_{0}+\beta_{1}\;{\text{micro-level knowledge}}_{\left(\text{fall or spring}\right)}\\ \;+\beta_{2}\;{\text{meso-level knowledge}}_{\left(\text{fall or spring}\right)} \end{array} \end{array}$$ (3)

### Contributions of early and late knowledge to subsequent influence

Because fall meso-level knowledge robustly predicted rising influence between the fall and spring, but only micro-level knowledge in the spring predicted current influence, our results suggest that influence may be related to the combination of different types of knowledge at different stages in network formation (i.e., first rapidly developing meso-level knowledge and then later fine-tuning micro-level knowledge). To assess this possibility, we directly tested the joint effect of early meso-level and late micro-level knowledge on spring influence (Fig. 5C and Table 3). To do so, we ran a linear regression model at the group level that predicts spring influence using the interaction of fall meso-level knowledge and spring micro-level knowledge (i.e., the beta coefficients from the subject-level logistic regressions, described above; Eq. 4)

$$\begin{array}{c} \begin{array}{c} \begin{array}{c} {\text{influence}}_{\left(\text{spring}\right)}∼\beta_{0}+\beta_{1}\;{\text{meso-level knowledge}}_{\left(\text{fall}\right)}+\beta_{2}\;{\text{micro-level knowledge}}_{\left(\text{spring}\right)}\\ \;+\beta_{3}\left({\text{meso-level knowledge}}_{\left(\text{fall}\right)}\times {\text{micro-level knowledge}}_{\left(\text{spring}\right)}\right) \end{array} \end{array} \end{array}$$ (4)

To test the robustness of this effect, we ran an additional model predicting spring influence, which used as predictors all four possible combinations of early and late network knowledge: fall micro × spring micro, fall micro × spring meso, fall meso × spring micro, and fall meso × spring meso.

## Supplementary Materials

### This PDF file includes:

Supplementary Text Figs. S1 to S5 Tables S1 to S4 References
