# Supplementary material for: Early insight into social network structure predicts climbing the social ladder
Source: Sci Adv. 2025 Jun 20;11(25):eads2133. doi: 10.1126/sciadv.ads2133 (PMC12180513; doi:10.1126/sciadv.ads2133)
Supplement: Supplementary file 1 — Supplementary Text Figs. S1 to S5 Tables S1 to S4 References [file sciadv.ads2133_sm.pdf]

Science Advances
AAAS

Supplementary Materials for

Early insight into social network structure predicts climbing the social ladder

Isabella C. Aslarus *et al.*

Corresponding author: Oriel FeldmanHall, oriel.feldmanhall@brown.edu

Sci. Adv. **11**, eads2133 (2025)
DOI: 10.1126/sciadv.ads2133

This PDF file includes:

Supplementary Text
Figs. S1 to S5
Tables S1 to S4
References

Supplementary Text

Supplementary methods for Friendship Surveys

In our Friendship Surveys, each subject reported their friendship status with every other subject, enabling us to map the veridical social network. To reduce the potential of social desirability biases in subjects' responses, we explicitly reminded subjects that their responses were anonymous at the start of every Friendship Survey, making it unlikely that subjects were motivated by the possibility that their responses might become linked to their identities or that others might find out what they had reported. To reduce the noise that could be introduced by friendships where one individual overstates their relationship while the other understates the connection, we limited our analyses to reciprocal friendships (i.e., friendships mutually reported by both individuals in a given dyad). We consider reciprocity a sufficient criterion to be confident that the surviving friendships documented in our work are similarly perceived by both parties. To reduce the potential that subjects' responses reflect differing personal interpretations of friendship, we provided a definition of friendship to all subjects at the start of every Friendship Survey, which read: "Your friends are defined as the people with whom you like to spend your free time. Since you arrived at Brown, who are the people you have been with most often for informal social activities, such as going out to lunch, dinner, drinks, films, visiting one another's rooms, and so on?" This shared definition of friendship, which revolves around concrete activities, reduces the possibility of more heterogeneous interpretations of friendship. Additionally, we note that modified versions of this specific definition of friendship—with the same or similar wording—have been used extensively in the social network science literature (e.g., *15*, *31*, *75-77*). Finally, to reduce the possibility of memory biases from forgetting potential friendships, we chose to generate friendship nominations via recognition, rather than via free recall. In every Friendship Survey, each subject was presented with the face and name of every other subject (much like a yearbook or a roster), thus ensuring that each subject considered and responded to all possible friendship nominees at every time point. There were no limitations on the number of friendships that could be reported, nor on the amount of time that could be taken to complete the survey.

Supplementary methods for the Network Knowledge Task

We note that the Network Knowledge Task was administered as part of a battery of behavioral tasks, which are not analyzed in this paper. We also note that, in the Spring administration of the Network Knowledge Task, we introduced a slight modification to the sampling method (i.e., the manner in which we custom-selected 30 other individuals from the network to serve as stimuli for each subject). In the Fall, samples were tailored to each subject to include approximately five of the subjects' friends, 10 friends-of-friends, and 15 friends-of-friends-of friends. In the Spring, we included the same four individuals in every subject's sample, while the remaining sample members were custom-selected for each subject using the same procedure as in the Fall. The four individuals who were included in all subjects' samples were popular individuals from different parts of the social network. This modification to the sampling procedure was in support of a separate research question (not addressed in this paper). Because our sampling procedure was already designed to include individuals at various distances from the subject, the inclusion of

these four specific individuals did not meaningfully alter the composition of each subject's sample.

Network similarity as a function of time elapsed

The time elapsed between adjacent network measurements (i.e., Friendship Surveys) was not always the same due to data collection constraints. For instance, the Network Knowledge Task was administered between Times 2 and 3, but not between Times 1 and 2, leading to longer time elapsed between the distribution of Friendship Surveys at Times 2 and 3 (47 days) vs. Times 1 and 2 (15 days). To support our finding of increasing network stability over time (Fig. 1C) while additionally accounting for the fact that adjacent Friendship Surveys were not equidistantly spaced, we statistically tested whether network similarity (quantified by Jaccard similarity) is related to the number of days elapsed between measurements. We found that Jaccard similarity and elapsed days are indeed significantly correlated ($r(13) = -0.83$, $p < 0.001$; Fig. S1B). While the network snapshots become increasingly dissimilar with greater time elapsed, we also observe even greater dissimilarity among the Fall networks (Fig. S1B, blue), and relatively greater similarity among the Spring networks (Fig. S1B, red), supporting the finding that the social network is initially quite unstable when students first arrive on campus and do not know one another, but stabilizes over time—i.e., any two network measurements separated by similar time intervals were more similar in the Spring than in the Fall.

Correlation between centrality measures (influence and friend count) across time

Influence and friend count were correlated in both the Fall (Fig. S2A; Spearman's rho = 0.92, $p < 0.001$) and the Spring (Fig. S2C; Spearman's rho = 0.88, $p < 0.001$). In addition, changes in influence and friend count between the Fall and Spring were correlated (Fig. S2B; Spearman's rho = 0.70, $p < 0.001$). While it is to be expected that different centrality measures are highly correlated (*78*), they are nevertheless associated with distinct outcomes in empirical research (*11*, *14*, *24*, *39*), including our findings. This aligns with theoretical work highlighting how different centrality measures are derived from different structural properties of a network, which suggests that they may confer different types of social advantages (*4*, *79*).

Correcting for skewness in centrality measures

Because both influence and friend count are right-skewed (see Fig. S2A and Fig. S2C), we ran exploratory analyses to replicate our main results using transformed centrality measures. We log-transformed friend count, and we logit-transformed influence (influence is bounded between 0 and 1, which would lead to large and strictly negative values if log-transforming). As with our main analyses, we calculated the change in influence and friend count as the difference between these transformed values between the Fall and Spring. Below, we report results replicating our main analyses with these log-/logit-transformed centrality measures.

Replicating our finding that early meso-level knowledge predicts rising influence over time, we found that changes in logit-transformed influence were predicted by more accurate meso-level knowledge in the Fall ($\beta = 0.62$, 95% CI = [0.06, 1.18], $p = 0.031$), but not micro-level knowledge ($\beta = -0.01$, 95% CI = [-0.74, 0.73], $p = 0.985$). Neither micro-level ($\beta = 0.05$, 95% CI = [-0.06, 0.17], $p = 0.360$) nor meso-level ($\beta = 0.01$, 95% CI = [-0.08, 0.10], $p = 0.841$) knowledge predicted changes in log-transformed friend count.

Consistent with our finding that network representation is not related to centrality in the unstable early network, we found that neither micro-level nor meso-level knowledge in the Fall predicted current logit-transformed influence (micro β = -0.17, 95% CI = [-0.53, 0.19], $p = 0.355$; meso β = 0.15, 95% CI = [-0.14, 0.43], $p = 0.307$) or log-transformed friend count (micro β = -0.09, 95% CI = [-0.26, 0.08], $p = 0.284$; meso β = 0.09, 95% CI = [-0.04, 0.22], $p = 0.174$) in the Fall.

Replicating our finding that influence in a stable network is associated with micro-level knowledge, we found that micro-level knowledge in the Spring predicts current logit-transformed influence (β = 0.97, 95% CI = [0.23, 1.71], $p = 0.011$). In addition, we found an association between current logit-transformed influence and meso-level knowledge in the Spring (β = 0.57, 95% CI = [0.004, 1.13], $p = 0.048$). We did not find a significant association between current log-transformed friend count and network knowledge in the Spring (micro β = 0.24, 95% CI = [-0.06, 0.55], $p = 0.115$; meso β = 0.23, 95% CI = [-0.001, 0.46], $p = 0.051$). When predicting logit-transformed influence in the Spring using both early meso-level and late micro-level representation, we did not find as strong an effect as in our original analyses, although the interaction between Fall meso-level and Spring micro-level knowledge was trending in the expected direction (β = 0.58, 95% CI = [-0.05, 1.20], $p = 0.071$).

Correlation between micro-level and meso-level knowledge across time

Micro-level and meso-level knowledge were not correlated in the Fall (Fig. S3A; $r(93) = -0.10$, $p = 0.359$) or the Spring (Fig. S3B; $r(75) = 0.18$, $p = 0.117$), further illustrating that these are two distinct types of knowledge about one's network.

Subjects exhibit insight into others' eigenvector centrality

We verified that eigenvector centrality (our measure of influence, derived quantitatively from network structure using Friendship Survey data) also maps onto subjects' more subjective perceptions of their peers, thus demonstrating that eigenvector centrality is a qualitatively meaningful feature of the individuals in the social network. We reason that, if members of a social network have insight into each other's eigenvector centrality, then their subjective perceptions of other individuals' friendships (as reported in the Network Knowledge Task, Fig. 1A in the main text and recreated in Fig. S4A) should be shaped by the eigenvector centrality of those individuals (for exact model specification, see Fig. S4B). We observe that subjects are most likely to guess that two individuals are friends when both individuals in the dyad have high eigenvector centrality (Fig. S4C; person 1 influence × person 2 influence β = 5.24, 95% CI = [4.38, 6.10], $p < 0.001$). This pattern of results holds when controlling for whether the two individuals actually reported being friends with each other, as well as the friend count of both individuals in the dyad (person 1 influence × person 2 influence β = 4.36, 95% CI = [3.29, 5.43], $p < 0.001$). These results illustrate that subjects are sensitive to our selected metric of influence (eigenvector centrality), to the extent that it biases their subjective perceptions of the network's structure. Consistent with previous research showing that eigenvector centrality aligns with subjective perceptions of influence (*30*), we demonstrate a clear link between this quantitative network metric and subjective network perceptions in our data, validating the meaningful role of eigenvector in shaping how subjects perceive each other.

Early meso-level knowledge predicts later influence for a simulated 'treatment' group matched on early influence with a 'control' group

To verify the robustness of the finding that early meso-level knowledge is associated with greater influence over time, we conducted an additional analysis by dividing our subjects into two matched groups using the R package *MatchIt* (*80*). The logic of the analysis is to create two groups that differ in their Fall-semester meso-level knowledge, but that are approximately matched on their Fall influence. If meso-level knowledge truly predicts rising influence in Spring—as the results in the main text suggest—then the group with higher meso-level knowledge in Fall should have higher influence in Spring. Therefore, the group with low meso-level knowledge simulates a 'control' group, while the group with high meso-level knowledge simulates a 'treatment' group.

We first performed manipulation checks to ensure that the matching procedure worked as intended (i.e., that the control and treatment groups differed on Fall meso-level knowledge, but did not differ on Fall influence). We used Welch's t-test to verify that Fall influence did not differ significantly between the control group ($M = 0.23$, $SD = 0.22$) and the treatment group ($M = 0.25$, $SD = 0.18$, $t(89.07) = -0.500$, $p = 0.618$; Fig. S5A). As specified by the matching procedure, Fall meso-level knowledge was significantly lower for the control group ($M = 0.42$, $SD = 0.51$) compared to the treatment group ($M = 2.25$, $SD = 0.83$, $t(76.41) = -12.972$, $p < 0.001$; Fig. S5B). Although the matching procedure did not explicitly account for micro-level knowledge, we also observed that micro-level knowledge did not differ between the control group ($M = 1.30$, $SD = 0.96$) and the treatment group ($M = 1.15$, $SD = 0.83$, $t(88.59) = 0.804$, $p = 0.424$; Fig. S5C). This incidental result is encouraging, as it increases our confidence in ascribing any differences between the control and treatment groups to differences in meso-level knowledge alone.

We next tested our key hypothesis that greater meso-level knowledge in Fall would predict greater influence in Spring. To do so, we used Welch's t-test to test whether Spring influence differed between the control and treatment groups. We observed significantly lower Spring influence for the control group ($M = 0.16$, $SD = 0.17$) as compared to the treatment group ($M = 0.32$, $SD = 0.32$, $t(64.52) = -2.921$, $p = 0.005$; Fig. S5D). In addition to the statistically significant difference in means between the control and treatment group, we observed that the median Spring influence was lower in the control group ($Mdn = 0.11$) as compared to the treatment group ($Mdn = 0.19$). We performed a 10,000-iteration permutation test on the difference in the medians of the control and treatment groups, which revealed marginally lower median Spring influence in the control group ($p = 0.081$). Although not significant, the direction of this result is consistent with our two other tests of statistical significance. Thus, by leveraging matching techniques to simulate a control group and a treatment group, we find converging evidence that possessing greater meso-level knowledge in Fall predicts significantly greater influence over time.

To further test the specificity of our hypothesis (i.e., that increasing influence uniquely depends on early meso-level knowledge and does not depend on micro-level knowledge), we tested whether low vs. high micro-level knowledge in Fall predicts later influence. Following the same procedure as the previous matching analysis, we created control and treatment groups that were matched on Fall influence but differed on Fall micro-level knowledge (low vs. high). We reasoned that, if meso-level knowledge is uniquely associated with increasing influence, then Spring influence should not differ between the new control and treatment groups. First, we

performed manipulation checks to verify the success of the matching procedure. Although the control and treatment groups were designed to be matched on Fall influence, we observed higher Fall influence in the control group (*M* = 0.29, *SD* = 0.23) as compared to the treatment group (*M* = 0.20, *SD* = 0.18, *t*(86.96) = 2.092, *p* = 0.040). This suggests that the correlation between higher influence and lower micro-level knowledge in the Fall (Fig. 5A) makes it difficult to create two groups that are perfectly matched on influence but differ on micro-level knowledge. As specified by the matching procedure, we observed significantly lower micro-level knowledge in the control group (*M* = 0.57, *SD* = 0.47) as compared to the treatment group (*M* = 1.92, *SD* = 0.67, *t*(82.33) = -11.240, *p* < 0.001). We also observed no significant difference in meso-level knowledge between the control group (*M* = 1.50, *SD* = 1.22) and the treatment group (*M* = 1.18, *SD* = 1.05, *t*(89.62) = 1.366, *p* = 0.175). Finally, in support of our hypothesis, we found no significant difference in Spring influence between the control group (*M* = 0.24, *SD* = 0.26) and the treatment group (*M* = 0.23, *SD* = 0.27, *t*(85.18) = 0.213, *p* = 0.832).

<u>Replication of results at a third timepoint in late Spring</u>

We collected a third wave of the network knowledge task (*N* = 80) in the late Spring (end of April), which was associated with the ground-truth social network constructed from friendship survey data collected at the fifth timepoint (early April). Using this data, we calculated centrality in the late Spring and change in centrality between the Fall and the late Spring. Below, we report results replicating our main analyses using data from the late Spring timepoint.

First, we replicated our finding that early meso-level knowledge predicts rising influence over time. Changes in influence between the Fall and the late Spring were predicted by meso-level knowledge in the Fall (β = 0.06, 95% CI = [0.01, 0.11], *p* = 0.015), but not micro-level knowledge (β = 0.06, 95% CI = [-0.001, 0.12], *p* = 0.054), an effect that remains robust when controlling for extroversion and Fall friend count (meso β = 0.06, 95% CI = [0.01, 0.11], *p* = 0.019; micro β = 0.06, 95% CI = [-0.01, 0.12], *p* = 0.079). As before, neither micro-level (β = 0.65, 95% CI = [-0.29, 1.59], *p* = 0.172) nor meso-level (β = 0.20, 95% CI = [-0.54, 0.95], *p* = 0.589) knowledge in the Fall predicted changes in friend count between the Fall and the late Spring.

Replicating our finding that influence in the early Spring is associated with micro-level knowledge, we find that influence in the late Spring is predicted by current micro-level (β = 0.10, 95% CI = [0.02, 0.19], *p* = 0.016) but not meso-level (β = 0.01, 95% CI = [-0.04, 0.07], *p* = 0.683) knowledge. We find no association between representation and friend count in the late Spring (micro β = 1.04, 95% CI = [-0.30, 2.37], *p* = 0.126; meso β = 0.16, 95% CI = [-0.70, 1.02], *p* = 0.715). In line with our analysis of the early Spring timepoint, we found a trending interaction between Fall meso-level representation and late Spring micro-level representation predicting influence in the late Spring (β = 0.07, 95% CI = [-0.001, 0.14], *p* = 0.052).

<u>Relationship between personality measures and network centrality</u>

To better understand how personality measures relate to network centrality (and changes in network centrality) in our sample, we tested all Big Five personality measures (openness, conscientiousness, extroversion, agreeableness, and neuroticism) as predictors of influence and friend count in both the Fall and Spring, as well as changes in influence and friend count over time (Table S1). Unsurprisingly, we find that extroversion significantly predicts both influence

and friend count in the Fall (influence β = 0.01, 95% CI = [0.002, 0.01], $p = 0.006$; friend count β = 0.27, 95% CI = [0.14, 0.41], $p < 0.001$) and Spring (influence β = 0.01, 95% CI = [0.005, 0.02], $p < 0.001$; friend count β = 0.22, 95% CI = [0.10, 0.33], $p < 0.001$). In the Spring, but not the Fall, greater neuroticism also predicts greater influence (β = 0.01, 95% CI = [0.002, 0.01], $p = 0.012$) and friend count (β = 0.15, 95% CI = [0.02, 0.28], $p = 0.024$). However, no personality measure predicts changes in influence or friend count over time (all p values > 0.1).

Next, we included personality measures as control variables in our main analyses that link micro- and meso-level knowledge to both current network centrality and changes in network centrality over time. We first re-ran all relevant analyses with only extroversion as a control variable, given that extroversion has the strongest theoretical connection to network centrality, and given that our empirical results (Table S1) demonstrate that extroversion is the most consistent and strongest predictor of network centrality in our dataset. Results (Table S2) recapitulate our main findings: compared to the findings reported in the main text, we find no differences in the direction or (non-)significance of the effects of micro- and meso-level knowledge on changes in influence, changes in friend count, Fall influence, Fall friend count, Spring influence, and Spring friend count. To highlight a key replication, we show that in the Fall, meso-level knowledge is the only predictor of increasing influence over time (β = 0.08, 95% CI = [0.03, 0.14], $p = 0.002$), with no significant effect of micro-level knowledge (β = 0.06, 95% CI = [-0.01, 0.12], $p = 0.075$) or extroversion (β = 0.003, 95% CI = [-0.01, 0.01], $p = 0.501$). Furthermore, in the Spring, current influence is predicted by micro-level knowledge (β = 0.13, 95% CI = [0.03, 0.22], $p = 0.011$) and extroversion (β = 0.01, 95% CI = [0, 0.02], $p = 0.043$), with no significant effects of meso-level knowledge (β = 0.06, 95% CI = [-0.01, 0.13], $p = 0.087$).

Aside from extroversion, the only other personality measure that predicted any network centrality measure at any timepoint was neuroticism, which predicted only Spring influence and Spring friend count (Table S1). We therefore re-ran our models predicting Spring influence and Spring friend count using Spring knowledge, with neuroticism added to the models as a control variable. Replicating the analyses reported in the main text, we find that Spring influence is predicted by Spring micro-level knowledge (β = 0.13, 95% CI = [0.03, 0.23], $p = 0.010$) but not Spring meso-level knowledge (β = 0.05, 95% CI = [-0.02, 0.13], $p = 0.155$), with no significant effect of neuroticism (β = 0.002, 95% CI = [-0.01, 0.01], $p = 0.634$). Further replicating our original analyses, neither micro- nor meso-level knowledge nor neuroticism predict Spring friend count (all p values > 0.2).

<u>Replication of results using individual community detection algorithms</u>
As discussed in the Methods (pages 17-18), we identify communities by taking the intersection of community assignments from four distinct community detection algorithms. We do so because the resulting 'consensus' communities are robust to and agnostic about the specific algorithm used, given that each community emerges independently under different algorithms. Here, we additionally confirm that our main findings do not hinge on a single clustering approach. To do so, we re-ran our main analyses using the communities identified by each of the four community detection algorithms used in the manuscript, listed here in order of their prevalence in the field of network science (based on Google Scholar citation counts at the time of this writing): 'edge betweenness' (over 20,000 citations; *69*), 'fast greedy' (nearly 10,000

citations; *70*), ‘infomap’ (over 5,000 citations; *53*, *68*), and lastly ‘walktrap’ (under 3,000 citations; *71*).

We only included communities with at least five members in further analyses, since this was the same threshold we used to identify ‘consensus’ communities. Results (Table S4) largely replicate the findings reported in the main text, which are based on ‘consensus’ communities. Two out of four community detection algorithms (‘edge betweenness’ and ‘infomap’) replicate our original findings in which we used ‘consensus’ communities: (1) while both micro- and meso-level knowledge predict rising influence, (2) only meso-level knowledge remains predictive when controlling for extroversion and Fall friend count, and (3) neither type of knowledge predicts changes in friend count. The ‘fast greedy’ algorithm largely replicates these effects, with the exception that micro-level knowledge remains predictive (p = 0.049) of increasing influence when controlling for extroversion and Fall friend count. Only the ‘walktrap’ algorithm—which is the least prevalent in the field—produces a different pattern of results. When using ‘walktrap’, only micro-level knowledge predicts increasing influence (regardless of control variables), while the effects of either knowledge type on changes in friend count remain null. While we emphasize our reasons for selecting ‘consensus’ communities over any one community detection algorithm, we also note that each of the field’s most commonly-used community detection algorithms produce a similar pattern of results.

Fig. S1.

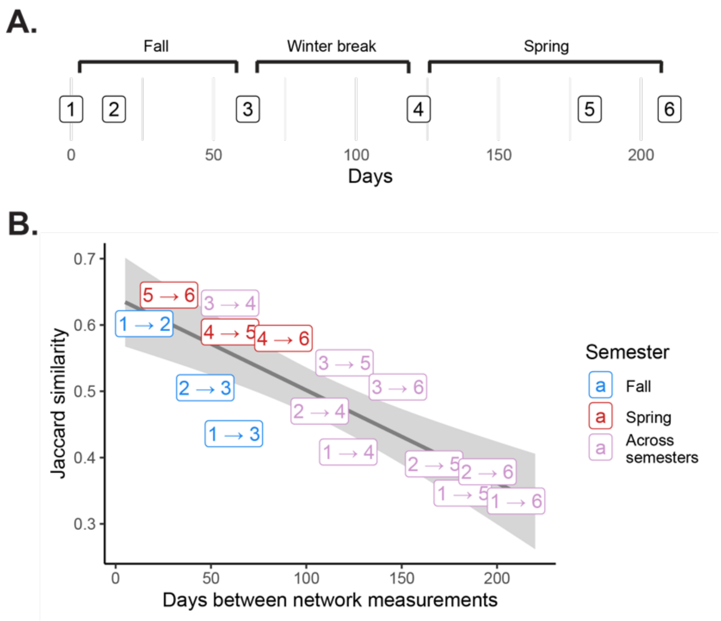

Fig. S1. Network snapshots become more dissimilar with greater time elapsed. (A) Timeline of friendship survey distribution, showing time interval in days between each network measurement. **(B)** As expected, the more days passed between a given pair of network measurements, the lower the Jaccard similarity of those two snapshots of the network. In support of the finding that network stability increased over time, snapshots of the early network in Fall (blue) are generally more dissimilar than snapshots of the late network in Spring (red).

Fig. S2.

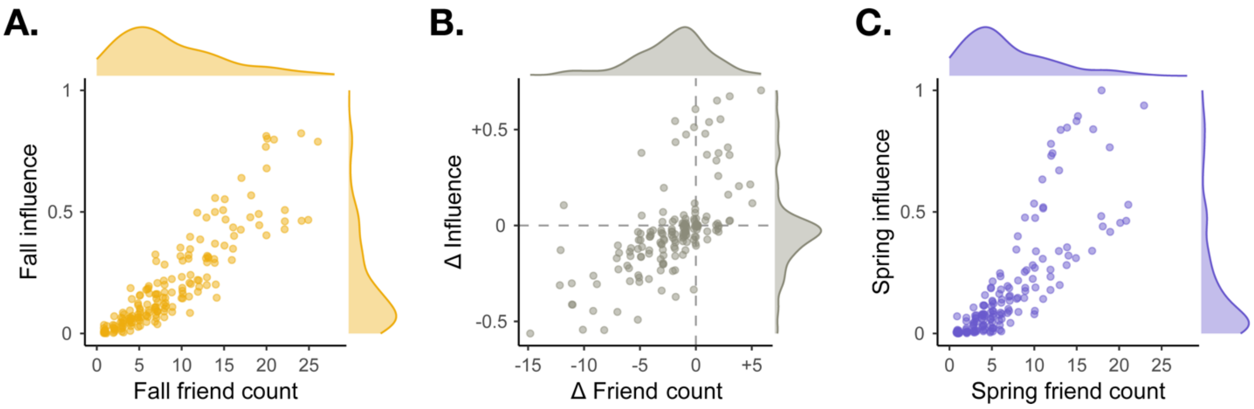

Fig. S2. Relationship between influence and friend count over time. A. Correlation between influence and friend count in the Fall. **B.** Correlation between change in influence and friend count between the Fall and the Spring. **C.** Correlation between influence and friend count in the Spring.

Fig. S3.

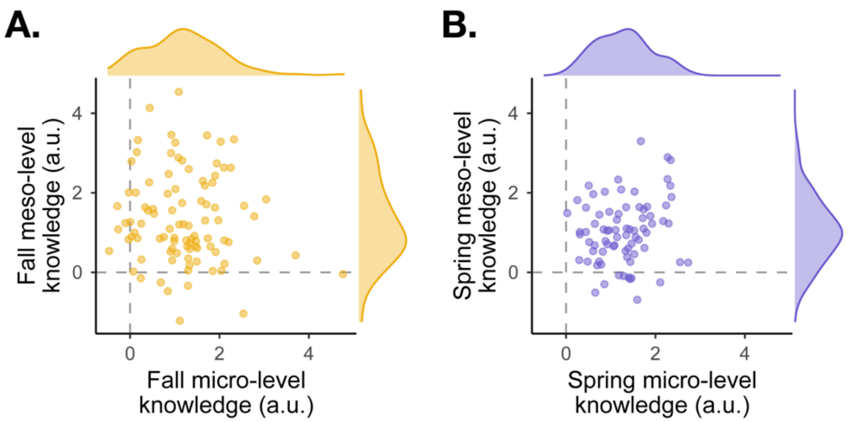

Fig. S3. Correlation between distinct sources of network knowledge over time. A & B. In both the Fall (panel A) and the Spring (panel B), micro-level knowledge and meso-level knowledge are not correlated.

Fig. S4.

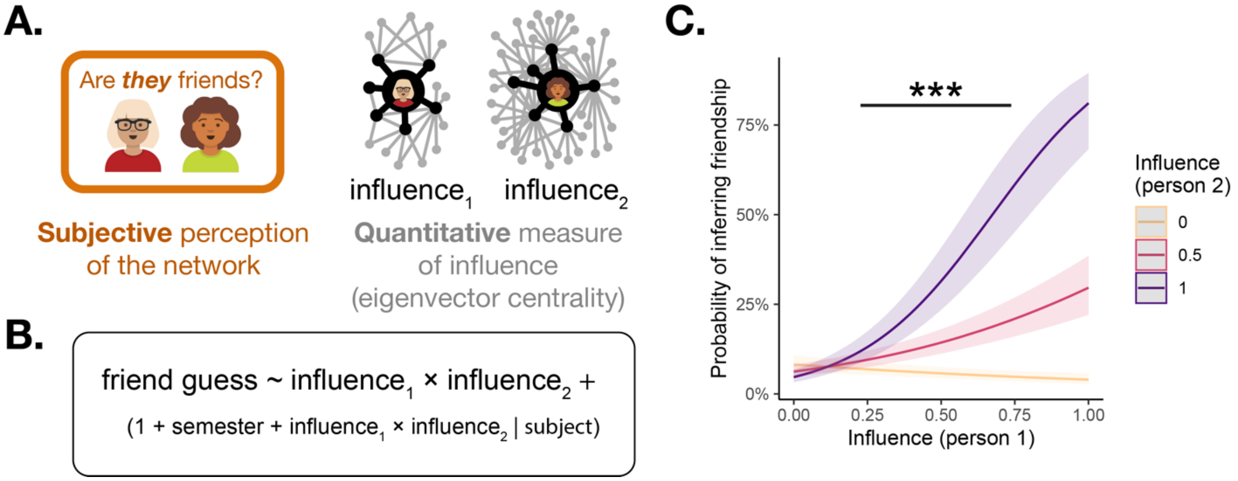

Fig. S4. Friendship inferences reveal that subjective perceptions of friendship are biased by quantitative measures of influence (eigenvector centrality). (A) We leveraged the Network Knowledge Task to explore whether subjects are sensitive to others' eigenvector centrality (a quantitative metric of influence derived from veridical network structure). **(B)** Model specification used to test whether subjects' inferences about the friendship status of a pair of individuals can be predicted by those individuals' eigenvector centrality. **(C)** Eigenvector centrality significantly predicted subjective friendship inferences, with subjects inferring that friendships are most likely between individuals with high influence/eigenvector centrality. Error ribbons represent the 95% confidence interval. * $p < 0.05$, ** $p < 0.01$, *** $p < 0.001$

Fig. S5.

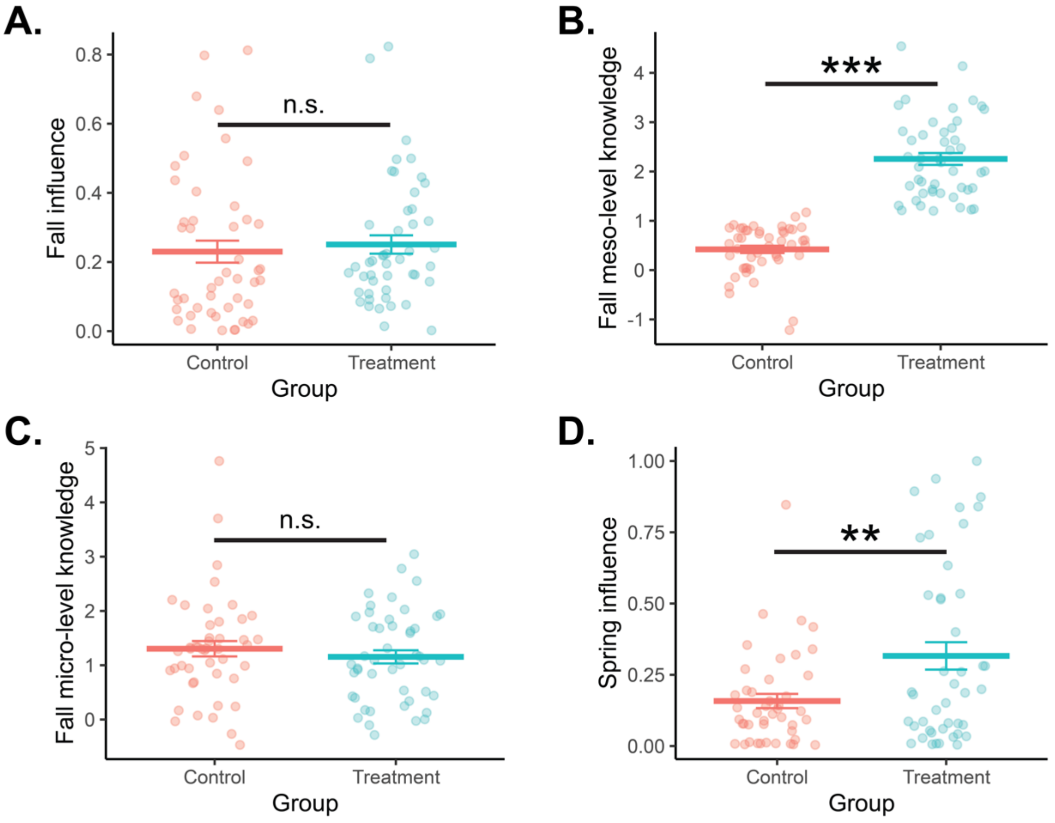

Fig. S5. Early meso-level knowledge predicts greater Spring influence in groups matched for Fall influence. (A) Simulated control and treatment groups, created by a matching procedure, show no difference in Fall influence. **(B)** Meso-level knowledge is higher for the treatment group. **(C)** The control and treatment groups, though not matched on micro-level knowledge, incidentally show no significant difference in this measure. **(D)** The treatment group, which had greater meso-level knowledge in Fall, exhibits greater influence in Spring compared to the control group. Crossbars represent the mean across subjects. Error bars represent one standard error. * $p < 0.05$, ** $p < 0.01$, *** $p < 0.001$

Table S1.

**Outcome variable**	**Predictor**	**β**	**95% confidence interval (lower, upper)**	***p*-value**
Fall influence ~ $\beta_0 + \beta_1$ openness + β_2 conscientiousness + β_3 extroversion + β_4 agreeableness + β_5 neuroticism				
Fall influence	Intercept	-0.14	(-0.47, 0.18)	0.384
	Openness	0.001	(-0.004, 0.01)	0.662
	Conscientiousness	-0.001	(-0.01, 0.004)	0.741
	Extroversion	0.01	(0.002, 0.01)	0.006**
	Agreeableness	0.003	(-0.003, 0.01)	0.344
	Neuroticism	0.003	(-0.002, 0.01)	0.300
Fall friend count ~ $\beta_0 + \beta_1$ openness + β_2 conscientiousness + β_3 extroversion + β_4 agreeableness + β_5 neuroticism				
Fall friend count	Intercept	-1.38	(-10.95, 8.19)	0.777
	Openness	0.01	(-0.14, 0.16)	0.943
	Conscientiousness	-0.10	(-0.25, 0.05)	0.202
	Extroversion	0.27	(0.14, 0.41)	8.2e-5***
	Agreeableness	0.09	(-0.09, 0.26)	0.335
	Neuroticism	0.11	(-0.03, 0.26)	0.133
Spring influence ~ $\beta_0 + \beta_1$ openness + β_2 conscientiousness + β_3 extroversion + β_4 agreeableness + β_5 neuroticism				
Spring influence	Intercept	-0.16	(-0.55, 0.24)	0.430
	Openness	-0.001	(-0.01, 0.005)	0.675
	Conscientiousness	-0.01	(-0.01, 0.001)	0.079
	Extroversion	0.01	(0.005, 0.02)	2.7e-4***
	Agreeableness	0.003	(-0.004, 0.01)	0.440
	Neuroticism	0.01	(0.002, 0.01)	0.012*
Spring friend count ~ $\beta_0 + \beta_1$ openness + β_2 conscientiousness + β_3 extroversion + β_4 agreeableness + β_5 neuroticism				
Spring friend count	Intercept	-5.74	(-14.08, 2.60)	0.176
	Openness	0.02	(-0.11, 0.15)	0.752
	Conscientiousness	-0.06	(-0.19, 0.07)	0.369
	Extroversion	0.22	(0.10, 0.33)	3.2e-4***

	Agreeableness	0.12	(-0.03, 0.27)	0.110
	Neuroticism	0.15	(0.02, 0.28)	0.024*
Change in influence ~ β_0 + β_1 openness + β_2 conscientiousness + β_3 extroversion + β_4 agreeableness + β_5 neuroticism				
Δ Influence	Intercept	-0.02	(-0.44, 0.39)	0.912
	Openness	-0.003	(-0.01, 0.004)	0.402
	Conscientiousness	-0.005	(-0.01, 0.002)	0.135
	Extroversion	0.005	(-0.001, 0.01)	0.106
	Agreeableness	0	(-0.01, 0.01)	0.937
	Neuroticism	0.01	(-0.001, 0.01)	0.123
Change in friend count ~ β_0 + β_1 openness + β_2 conscientiousness + β_3 extroversion + β_4 agreeableness + β_5 neuroticism				
Δ Friend count	Intercept	-5.03	(-11.59, 1.53)	0.132
	Openness	0.01	(-0.09, 0.11)	0.837
	Conscientiousness	0.04	(-0.07, 0.14)	0.506
	Extroversion	-0.04	(-0.13, 0.06)	0.441
	Agreeableness	0.05	(-0.07, 0.17)	0.406
	Neuroticism	0.03	(-0.08, 0.13)	0.626

Table S1. Personality trait predictors of influence and friend count across time. * $p < 0.05$, ** $p < 0.01$, *** $p < 0.001$

Table S2.

**Outcome variable**	**Predictor**	**β**	**95% confidence interval (lower, upper)**	***p*-value**
Change in influence ~ β_0 + β_1 Fall micro-level knowledge + β_2 Fall meso-level knowledge + β_3 extroversion				
Δ Influence	Intercept	-0.26	(-0.50, -0.03)	0.028*
	Fall micro	0.06	(-0.01, 0.12)	0.075
	Fall meso	0.08	(0.03, 0.14)	0.002**
	Extroversion	0.003	(-0.01, 0.01)	0.501
Change in friend count ~ β_0 + β_1 Fall micro-level knowledge + β_2 Fall meso-level knowledge + β_3 extroversion				
Δ Friend count	Intercept	-2.76	(-6.21, 0.68)	0.114
	Fall micro	0.63	(-0.31, 1.56)	0.188
	Fall meso	0.10	(-0.61, 0.81)	0.779
	Extroversion	-0.02	(-0.14, 0.10)	0.718
Fall influence ~ β_0 + β_1 Fall micro-level knowledge + β_2 Fall meso-level knowledge + β_3 extroversion				
Fall influence	Intercept	0.14	(-0.04, 0.32)	0.120
	Fall micro	-0.04	(-0.09, 0.005)	0.078
	Fall meso	-0.02	(-0.05, 0.02)	0.387
	Extroversion	0.01	(0, 0.01)	0.042*
Fall friend count ~ β_0 + β_1 Fall micro-level knowledge + β_2 Fall meso-level knowledge + β_3 extroversion				
Fall friend count	Intercept	5.00	(-0.04, 10.05)	0.052
	Fall micro	-1.08	(-2.40, 0.23)	0.106
	Fall meso	0.15	(-0.88, 1.18)	0.774
	Extroversion	0.23	(0.05, 0.40)	0.011*
Spring influence ~ β_0 + β_1 Spring micro-level knowledge + β_2 Spring meso-level knowledge + β_3 extroversion				
Spring influence	Intercept	-0.23	(-0.48, 0.02)	0.074
	Spring micro	0.13	(0.03, 0.22)	0.011*
	Spring meso	0.06	(-0.01, 0.13)	0.087
	Extroversion	0.01	(0, 0.02)	0.043*

Spring friend count ~ β_0 + β_1 Spring micro-level knowledge + β_2 Spring meso-level knowledge + β_3 extroversion				
Spring friend count	Intercept	1.30	(-3.98, 6.58)	0.626
	Spring micro	1.08	(-0.93, 3.09)	0.287
	Spring meso	0.91	(-0.60, 2.42)	0.235
	Extroversion	0.15	(-0.02, 0.32)	0.089

Table S2. Network centrality predicted by knowledge, controlling for extroversion. * $p < 0.05$, ** $p < 0.01$, *** $p < 0.001$

Table S3.

**Outcome variable**	**Predictor**	**β**	**95% confidence interval (lower, upper)**	***p*-value**
Spring influence ~ β_0 + β_1 Spring micro-level knowledge + β_2 Spring meso-level knowledge + β_3 neuroticism				
Spring influence	Intercept	-0.05	(-0.27, 0.17)	0.640
	Spring micro	0.13	(0.03, 0.23)	0.010*
	Spring meso	0.05	(-0.02, 0.13)	0.155
	Neuroticism	0.002	(-0.01, 0.01)	0.634
Spring friend count ~ β_0 + β_1 Spring micro-level knowledge + β_2 Spring meso-level knowledge + β_3 neuroticism				
Spring friend count	Intercept	4.83	(0.28, 9.38)	0.038*
	Spring micro	1.27	(-0.82, 3.35)	0.230
	Spring meso	0.78	(-0.78, 2.33)	0.321
	Neuroticism	0.02	(-0.17, 0.20)	0.872

Table S3. Spring network centrality predicted by knowledge, controlling for neuroticism. * $p < 0.05$, ** $p < 0.01$, *** $p < 0.001$

Table S4.

**Community detection algorithm**	**Outcome variable**	**Predictor**	**β**	**95% confidence interval (lower, upper)**	***p*-value**
Edge betweenness	*Change in influence ~ β_0 + β_1 Fall micro-level knowledge + β_2 Fall meso-level knowledge*				
	Δ Influence	Intercept	-0.19	(-0.30, -0.08)	7.5e-4***
		Fall micro	0.07	(0.01, 0.14)	0.034*
		Fall meso	0.07	(0.03, 0.12)	0.003**
	Change in influence ~ β_0 + β_1 Fall micro-level knowledge + β_2 Fall meso-level knowledge + β_3 Fall friend count + β_4 Fall extroversion				
	Δ Influence	Intercept	-0.21	(-0.44, 0.03)	0.085
		Fall micro	0.05	(-0.02, 0.12)	0.128
		Fall meso	0.08	(0.03, 0.13)	0.001**
		Fall friend count	-0.01	(-0.02, -0.001)	0.037*
		Extroversion	0.01	(-0.003, 0.01)	0.223
	Change in friend count ~ β_0 + β_1 Fall micro-level knowledge + β_2 Fall meso-level knowledge				
	Δ Friend count	Intercept	-3.31	(-4.95, -1.66)	1.4e-4***
		Fall micro	0.99	(0.02, 1.95)	0.045*
		Fall meso	-0.29	(-0.98, 0.40)	0.409
Infomap	*Change in influence ~ β_0 + β_1 Fall micro-level knowledge + β_2 Fall meso-level knowledge*				
	Δ Influence	Intercept	-0.20	(-0.31, -0.09)	3.8e-4***
		Fall micro	0.07	(0.01, 0.13)	0.030*
		Fall meso	0.08	(0.03, 0.12)	0.002**
	Change in influence ~ β_0 + β_1 Fall micro-level knowledge + β_2 Fall meso-level knowledge + β_3 Fall friend count + β_4 Fall extroversion				
	Δ Influence	Intercept	-0.21	(-0.44, 0.02)	0.073
		Fall micro	0.06	(-0.003, 0.12)	0.062
		Fall meso	0.08	(0.03, 0.12)	0.002**
		Fall friend count	-0.01	(-0.02, 0.002)	0.128
		Extroversion	0.004	(-0.004, 0.01)	0.375
	Change in friend count ~ β_0 + β_1 Fall micro-level knowledge + β_2 Fall meso-level knowledge				
	Δ Friend count	Intercept	-3.40	(-5.04, -1.76)	8.7e-5***
		Fall micro	0.68	(-0.28, 1.64)	0.161
		Fall meso	0.12	(-0.6, 0.84)	0.742

Fast greedy	*Change in influence ~ β_0 + β_1 Fall micro-level knowledge + β_2 Fall meso-level knowledge*				
	Δ Influence	Intercept	-0.21	(-0.32, -0.09)	6.9e-4***
		Fall micro	0.08	(0.01, 0.16)	0.025*
		Fall meso	0.06	(0.02, 0.11)	0.007**
	Change in influence ~ β_0 + β_1 Fall micro-level knowledge + β_2 Fall meso-level knowledge + β_3 Fall friend count + β_4 Fall extroversion				
	Δ Influence	Intercept	-0.19	(-0.43, 0.05)	0.124
		Fall micro	0.07	(0, 0.15)	0.049*
		Fall meso	0.07	(0.02, 0.11)	0.006**
		Fall friend count	-0.01	(-0.02, 0.001)	0.076
		Extroversion	0.003	(-0.01, 0.01)	0.465
	Change in friend count ~ β_0 + β_1 Fall micro-level knowledge + β_2 Fall meso-level knowledge				
	Δ Friend count	Intercept	-3.14	(-4.85, -1.43)	4.6e-4***
		Fall micro	0.82	(-0.27, 1.91)	0.140
		Fall meso	-0.29	(-0.95, 0.38)	0.394
Walktrap	*Change in influence ~ β_0 + β_1 Fall micro-level knowledge + β_2 Fall meso-level knowledge*				
	Δ Influence	Intercept	-0.20	(-0.32, -0.08)	9.9e-4***
		Fall micro	0.12	(0.05, 0.20)	9.4e-4***
		Fall meso	0.03	(-0.02, 0.08)	0.191
	Change in influence ~ β_0 + β_1 Fall micro-level knowledge + β_2 Fall meso-level knowledge + β_3 Fall friend count + β_4 Fall extroversion				
	Δ Influence	Intercept	-0.28	(-0.53, -0.04)	0.026*
		Fall micro	0.12	(0.05, 0.19)	0.002**
		Fall meso	0.03	(-0.02, 0.08)	0.268
		Fall friend count	-0.01	(-0.02, 0.003)	0.181
		Extroversion	0.01	(-0.002, 0.01)	0.152
	Change in friend count ~ β_0 + β_1 Fall micro-level knowledge + β_2 Fall meso-level knowledge				
	Δ Friend count	Intercept	-3.15	(-4.84, -1.46)	3.9e-4***
		Fall micro	0.80	(-0.24, 1.84)	0.132
		Fall meso	-0.30	(-1.00, 0.39)	0.393

Table S4. Changes in network centrality predicted by knowledge from different community detection algorithms. * $p < 0.05$, ** $p < 0.01$, *** $p < 0.001$

REFERENCES AND NOTES

1. P. Bourdieu "The forms of capital" in *Handbook of Theory and Research for the Sociology of Education*, J. Richardson, Ed. Greenwood, 1985, pp. 241–258.

2. R. A. Brands, Cognitive social structures in social network research: A review. *J. Organ. Behav.* **34**, S82–S103 (2013).

3. A. Banerjee, A. G. Chandrasekhar, E. Duflo, M. O. Jackson, The Diffusion of Microfinance. *Science* **341**, 1236498 (2013).

4. S. P. Borgatti, Centrality and network flow. *Soc. Netw.* **27**, 55–71 (2005).

5. T. W. Valente, Network interventions. *Science* **337**, 49–53 (2012).

6. T. W. Valente, P. Pumpuang, Identifying opinion leaders to promote behavior change. *Health Educ. Behav.* **34**, 881–896 (2007).

7. E. L. Paluck, H. Shepherd, P. M. Aronow, Changing climates of conflict: A social network experiment in 56 schools. *Proc. Natl. Acad. Sci. U.S.A.* **113**, 566–571 (2016).

8. E. Breza, A. G. Chandrasekhar, Social networks, reputation, and commitment: evidence from a savings monitors experiment. *Econometrica* **87**, 175–216 (2019).

9. E. M. Airoldi, N. A. Christakis, Induction of social contagion for diverse outcomes in structured experiments in isolated villages. *Science* **384**, eadi5147 (2024).

10. A. Banerjee, A. G. Chandrasekhar, E. Duflo, M. O. Jackson, Using gossips to spread information: Theory and evidence from two randomized controlled trials. *Rev. Econ. Stud.* **86**, 2453–2490 (2019).

11. G. A. Ballinger, R. Cross, B. C. Holtom, The right friends in the right places: Understanding network structure as a predictor of voluntary turnover. *J. Appl. Psychol.* **101**, 535–548 (2016).

12. R. Basyouni, C. Parkinson, Mapping the social landscape: tracking patterns of interpersonal relationships. *Trends Cogn. Sci.* **26**, 204–221 (2022).

13. D. Brewer, A note on the relationship between centrality and cultural knowledge in a professional network. *Connections* **15**, 21–28 (1992).

14. R. Burt, M. Kilduff, S. Tasselli, Social network analysis: Foundations and frontiers on advantage. *Annu. Rev. Psychol.* **64**, 527–547 (2013).

15. R. S. Burt, *Structural Holes: The Social Structure of Competition*. (Harvard Univ. Press, 1992).

16. L. Ellwardt, G. Labianca, R. Wittek, Who are the objects of positive and negative gossip at work?: A social network perspective on workplace gossip. *Soc Netw* **34**, 193–205 (2012).

17. M. Kilduff, D. Krackhardt, Bringing the Individual Back in: A Structural Analysis of the Internal Market for Reputation in Organizations. *Acad. Manage. J.* **37**, 87–108 (1994).

18. D. Krackhardt, D. J. Brass, "Intraorganizational networks: The micro side" in *Advances in social network analysis*, S. Wasserman, J. Galaskiewicz, Eds. (SAGE Publications; 1994), pp. 207–229.

19. L. Thiele, N. C. Sauer, S. Kauffeld, Why extraversion is not enough: the mediating role of initial peer network centrality linking personality to long-term academic performance. *High Educ.* **76**, 789–805 (2018).

20. D. Bondonio, Predictors of accuracy in perceiving informal social networks. *Soc Netw* **20**, 301–330 (1998).

21. E. Cappella, J. W. Neal, N. Sahu, Children's agreement on classroom social networks: Cross-level predictors in urban elementary schools. *Merrill-Palmer Quart.* **58**, 285–313 (2012).

22. T. Casciaro, Seeing things clearly: social structure, personality, and accuracy in social network perception. *Soc Netw* **20**, 331–351 (1998).

23. T. Casciaro, K. M. Carley, D. Krackhardt, Positive affectivity and accuracy in social network perception. *Motiv. Emot.* **23**, 285–306 (1999).

24. E. S. Lee, A. M. Lease, D. T. Robinson, S. Neuharth-Pritchett, J. Xu, Individual difference in perceptions of social structure: Social standing predicts accuracy in social network perception. *Soc. Develop.* **31**, 549–567 (2022).

25. F. J. Flynn, R. E. Reagans, E. T. Amanatullah, D. R. Ames, Helping one's way to the top: Self-monitors achieve status by helping others and knowing who helps whom. *J. Pers. Soc. Psychol.* **91**, 1123–1137 (2006).

26. D. Krackhardt, Assessing the political landscape: Structure, cognition, and power in organizations. *Adm. Sci. Q.* **35**, 342–369 (1990).

27. A. Mehra, A. L. Dixon, D. J. Brass, B. Robertson, the social network ties of group leaders: Implications for group performance and leader reputation. *Organ. Sci.* **17**, 64–79 (2006).

28. P. Bonacich, Power and centrality: A family of measures. *Am. J. Sociol.* **92**, 1170–1182 (1987).

29. P. Bonacich, Some unique properties of eigenvector centrality. *Soc Netw* **29**, 555–564 (2007).

30. A. Banerjee, A. Chandrasekhar, E. Duflo, M. Jackson, *Gossip: Identifying Central Individuals in a Social Network*. (National Bureau of Economic Research, 2014).

31. C. Parkinson, A. M. Kleinbaum, T. Wheatley, Spontaneous neural encoding of social network position. *Nat. Hum. Behav.* **1**, 1–7 (2017).

32. M. Yucel, G. R. Sjobeck, R. Glass, J. Rottman, Being in the know: Social network analysis of gossip and friendship on a college campus. *Hum. Nat.* **32**, 603–621 (2021).

33. P. D. Meo, K. Musial-Gabrys, D. Rosaci, G. M. L. Sarnè, L. Aroyo, Using centrality measures to predict helpfulness-based reputation in trust networks. *ACM Trans. Internet Technol.* **17**, 1–20 (2017).

34. P. Balkundi, M. Kilduff, The ties that lead: A social network approach to leadership. *Leader. Quart.* **17**, 419–439 (2006).

35. R. S. Burt, D. Ronchi, Teaching executives to see social capital: Results from a field experiment. *Soc. Sci. Res.* **36**, 1156–1183 (2007).

36. G. Janicik, R. Larrick, Social Network Schemes and the Learning of Incomplete Networks. *J. Pers. Soc. Psychol.* **88**, 348–364 (2005).

37. D. Krackhardt, Cognitive social structures. *Soc. Netw.* **9**, 109–134 (1987).

38. S. Van Waes, S. De Maeyer, N. M. Moolenaar, P. Van Petegem, P. Van den Bossche, Strengthening networks: A social network intervention among higher education teachers. *Learn. Instruct.* **53**, 34–49 (2018).

39. S. Lee, J. Foote, Z. Wittrock, S. Xu, L. Niu, D. C. French, Adolescents' perception of peer groups: Psychological, behavioral, and relational determinants. *Soc. Sci. Res.* **65**, 181–194 (2017).

40. M. E. Brashears, E. Quintane, The microstructures of network recall: How social networks are encoded and represented in human memory. *Soc. Netw.* **41**, 113–126 (2015).

41. L. C. Freeman, Filling in the blanks: A theory of cognitive categories and the structure of social affiliation. *Soc. Psychol. Q.* **55**, 118–127 (1992).

42. M. Kilduff, C. Crossland, W. Tsai, D. Krackhardt, Organizational network perceptions versus reality: A small world after all? *Organ. Behav. Hum. Decis. Process.* **107**, 15–28 (2008).

43. M. E. J. Newman, The structure and function of complex networks. *SIAM Rev.* **45**, 167–256 (2003).

44. D. J. Watts, S. H. Strogatz, Collective dynamics of 'small-world' networks. *Nature* **393**, 440–442 (1998).

45. C. Pomeroy, R. M. Bond, P. J. Mucha, S. J. Cranmer, Dynamics of social network emergence explain network evolution. *Sci. Rep.* **10**, 21876 (2020).

46. M. E. Brashears, Humans use compression heuristics to improve the recall of social networks. *Sci. Rep.* **3**, 1513 (2013).

47. L. C. Freeman, A. K. Romney, S. C. Freeman, Cognitive structure and informant accuracy. *Am. Anthropol.* **89**, 310–325 (1987).

48. C. Lynn, A. Kahn, D. Bassett, Structure from noise: Mental errors yield abstract representations of events. arXiv: 1805.12491 [q-bio.NC] (2018).

49. J. E. Marineau, G. Labianca, D. J. Brass, S. P. Borgatti, P. Vecchi, Individuals' power and their social network accuracy: A situated cognition perspective. *Soc. Netw.* **54**, 145–161 (2018).

50. J. W. Neal, Z. P. Neal, E. Cappella, Seeing and being seen: Predictors of accurate perceptions about classmates' relationships. *Soc. Netw.* **44**, 1–8 (2016).

51. B. Simpson, B. Markovsky, M. Steketee, Power and the perception of social networks. *Soc. Netw.* **33**, 166–171 (2011).

52. M. Rosvall, C. T. Bergstrom, An information-theoretic framework for resolving community structure in complex networks. *Proc. Natl. Acad. Sci. U.S.A.* **104**, 7327–7331 (2007).

53. M. Rosvall, C. T. Bergstrom, Maps of random walks on complex networks reveal community structure. *Proc. Natl. Acad. Sci. U.S.A.* **105**, 1118–1123 (2008).

54. C. E. Shannon, A mathematical theory of communication. *Bell Syst. Tech. J.* **27**, 379–423 (1948).

55. N. Slonim, G. S. Atwal, G. Tkačik, W. Bialek, Information-based clustering. *Proc. Natl. Acad. Sci. U.S.A.* **102**, 18297–18302 (2005).

56. D. Liben-Nowell, J. Kleinberg, "The link prediction problem for social networks," in *Proceedings of the Twelfth International Conference on Information and Knowledge Management* (Association for Computing Machinery, 2003), pp. 556–559.

57. J.-Y. Son, A. Bhandari, O. FeldmanHall, Cognitive maps of social features enable flexible inference in social networks. *Proc. Natl. Acad. Sci. U. S. A.* **118**, e2021699118 (2021).

58. J.-Y. Son, A. Bhandari, O. FeldmanHall, Abstract cognitive maps of social network structure aid adaptive inference. *Proc. Natl. Acad. Sci. U.S.A.* **120**, e2310801120 (2023).

59. O. FeldmanHall, J. E. Dunsmoor, A. Tompary, L. E. Hunter, A. Todorov, E. A. Phelps, Stimulus generalization as a mechanism for learning to trust. *Proc. Natl. Acad. Sci. U.S.A.* **115**, E1690–E1697 (2018).

60. S. J. Gershman, H. T. Pouncy, H. Gweon, Learning the structure of social influence. *Cognit. Sci.* **41**, 545–575 (2017).

61. M. K. Ho, D. Abel, T. L. Griffiths, M. L. Littman, The value of abstraction. *Curr. Opin. Behav. Sci.* **29**, 111–116 (2019).

62. T. Lau, H. T. Pouncy, S. J. Gershman, M. Cikara, Discovering social groups via latent structure learning. *J. Exp. Psychol. Gen.* **147**, 1881–1891 (2018).

63. R. N. Shepard, Toward a universal law of generalization for psychological science. *Science* **237**, 1317–1323 (1987).

64. M. Granovetter, The strength of weak ties. *Am. J. Sociol.* **78**, 1360–1380 (1973).

65. E. C. Baek, M. A. Porter, C. Parkinson, Social network analysis for social neuroscientists. *Soc. Cogn. Affect. Neurosci.* **16**, 883–901 (2021).

66. T. Pederson. tidygraph: A Tidy API for Graph Manipulation, version 1.3.1.9000 (2024); https://github.com/thomasp85/tidygraph, https://tidygraph.data-imaginist.com.

67. G. Csardi, T. Nepusz, The igraph software package for complex network research. *InterJournal Complex Syst.* **1695**, 1–9 (2006).

68. M. Rosvall, D. Axelsson, C. T. Bergstrom, The map equation. *Eur. Phys. J. Spec. Top.* **178**, 13–23 (2009).

69. M. Girvan, M. E. J. Newman, Community structure in social and biological networks. *Proc. Natl. Acad. Sci. U.S.A.* **99**, 7821–7826 (2002).

70. A. Clauset, M. E. J. Newman, C. Moore, Finding community structure in very large networks. *Phys. Rev. E* **70**, 066111 (2004).

71. P. Pons, M. Latapy, "Computing Communities in Large Networks Using Random Walks" in *Computer and Information Sciences - ISCIS 2005*, pInar Yolum, T. Güngör, F. Gürgen, C. Özturan, Eds. (Springer, 2005), pp. 284–293.

72. O. P. John, E. M. Donahue, R. L. Kentle, The Big Five Inventory, versions 4a and 54 (University of California, Berkeley, Institute of Personality and Social Research; 1991).

73. O. P. John, L. P. Naumann, C. J. Soto, "Paradigm shift to the integrative big five trait taxonomy," in *Handbook of personality: Theory and research*, O. P. John, R. W. Robins, L. A. Pervin, Eds. (Guilford Press, ed. 3, 2008), pp. 114–158.

74. D. Lüdecke, ggeffects: Tidy data frames of marginal effects from regression models. *J. Open Source Soft.* **3**, 772 (2018).

75. D. C. Feiler, A. M. Kleinbaum, Popularity, similarity, and the network extraversion bias. *Psychol. Sci.* **26**, 593–603 (2015).

76. E. C. Baek, R. Hyon, K. López, E. S. Finn, M. A. Porter, C. Parkinson, In-degree centrality in a social network is linked to coordinated neural activity. *Nat. Commun.* **13**, 1118 (2022).

77. B. Sievers, C. Welker, U. Hasson, A. M. Kleinbaum, T. Wheatley, Consensus-building conversation leads to neural alignment. *Nat. Commun.* **15**, 3936 (2024).

78. T. W. Valente, K. Coronges, C. Lakon, E. Costenbader, How correlated are network centrality measures? *Connect* **28**, 16–26 (2008).

79. M. O. Jackson, A typology of social capital and associated network measures. *Soc. Choice Welf* **54**, 311–336 (2020).

80. D. E. Ho, K. Imai, G. King, E. A. Stuart, MatchIt: Nonparametric preprocessing for parametric causal inference. *J. Stat. Softw.* **42**, 1–28 (2011).
